# Thermoresponsive Core-Shell Nanoparticles: Does Core Size Matter?

**DOI:** 10.3390/ma11091654

**Published:** 2018-09-07

**Authors:** Martina Schroffenegger, Erik Reimhult

**Affiliations:** University of Natural Resources and Life Sciences Vienna, Muthgasse 11, 1190 Vienna, Austria; martina.schroffenegger@boku.ac.at

**Keywords:** poly(2-isopropyl-2-oxazoline) (PiPOx), lower critical solution temperature (LCST), critical flocculation temperature (CFT), superparamagnetic iron oxide nanoparticles (SPION), thermoresponsive polymer, reversible nanoparticle aggregation, spherical polymer brush shell, dynamic scanning calorimetry (DSC), dynamic light scattering (DLS), size and curvature dependence, core-shell nanoparticle

## Abstract

Nanoparticles grafted with a dense brush of hydrophilic polymers exhibit high colloidal stability. However, reversible aggregation can be triggered by an increase in temperature if the polymer is thermoresponsive, as the polymer shell partly loses its hydration. We investigate the role of nanoparticle curvature on the critical solution temperature (CST) of grafted poly(2-isopropyl-2-oxazoline) (PiPOx) and critical flocculation temperature (CFT) of the core-shell nanoparticle dispersion. Cores with diameters ranging from 5 to 21 nm were studied by temperature-cycled dynamic light scattering and differential scanning calorimetry over a large range of concentrations. We show that core size and curvature only have a minor influence on particle aggregation (CFT and cluster size), while they have major influence on the CST of the polymer shell. The densely grafted shells exhibit three distinct solvation transitions, the relative contributions of each is controlled by the core curvature. We link these transitions to different polymer density regimes within the spherical brush and demonstrate that the CST of the innermost part of the brush coincides with the CFT of the particle dispersion.

## 1. Introduction

Nanoparticles with inorganic cores and polymer shells are an interesting class of composite materials. They combine the properties of both the core and the shell. Such core-shell nanoparticles dispersed in a liquid can show very complex behaviour due to the interplay between the properties of the core, polymer and continuous phase. This enables the creation of so-called smart multi-functional materials. The shell has a key function in such composites. It must stabilize the particles, in other words prevent and control aggregation. Core-shell particles dispersed in a solvent can change their aggregation state by a change in the solubility of the polymer shell. A solvated shell provides steric-osmotic repulsion that keeps the particle cores from aggregating. In polar solvents, such as water, dispersible polymers often show a lower critical solution temperature (LCST). Above the LCST the solubility of the polymer decreases drastically. 

Thermoresponsive colloidal smart materials received much attention in recent years, due to their many fields of application; they can be used for medical applications [1] such as drug delivery [2], hyperthermia [3] or as contrast agents [4], but also for catalysis [5], sensing [6] or separation [7]. To optimize such applications, it is crucial to know the phase diagram of the core-shell system under realistic conditions. Many parameters have an influence on the critical solution temperature (CST) of a polymer, such as the concentration [8], the end group [9], the monomer composition [10], the ionic strength of the aqueous surrounding [8] and, as recently demonstrated, the local monomer concentration determined by the polymer shell morphology [8,11] 

The phase behaviour of 1.3-nm-in-diameter gold nanoparticles grafted with poly(*N*-isopropylacrylamide) (PNIPAM) was previously investigated by Tenhu and co-workers [9,11,12]. The grafting process lowered the CST at a polymer concentration of 0.2 mmol by 2 °C [9]. Furthermore, the CST was studied over a concentration range from 1.1 wt % to 83.3 wt % by differential scanning calorimetry (DSC). The true LCST was found at a concentration of 43 wt % at 11.4 °C. Interestingly, Shan et al. identified two CST transitions within the shell using DSC. The transition at lower temperature was assigned to the inner shell, in which the polymers are densely packed and therefore less hydrated. The transition at higher temperature was assigned to an outer shell, which exhibits coil-like conformation and is highly hydrated [9]. 

Kurzhals et al. could show, that not only grafting has an impact on the polymer shell CST, but also the molecular weight (MW) of the grafted polymer matters [13]. Iron oxide nanoparticles with core sizes around 10 nm grafted with different MW of PNIPAM were studied. Nanoparticles with 5 kg mol^−1^ PNIPAM grafted to the core have the highest CST at 39.5 °C measured with DSC. On the other hand, particles with a PNIPAM MW of 30 kg mol^−1^ at similar grafting density showed a CST that was 4.3 °C lower. A similar study was performed on iron oxide nanoparticles grafted with poly(2-isopropyl-2-oxazoline) (PiPOx) [8], showing that the results could be generalized to LCST polymer systems beyond PNIPAM. The CST is observed at 40 °C at a concentration of 1 g L^−1^ if PiPOx with a MW of 6 kg mol^−1^ is grafted, while with a MW of 33 kg mol^−1^ it is lowered to 35 °C [8]. Furthermore, it was shown that grafting of PiPOx of higher MW (in this case 14 kg mol^−1^) led to the shell showing two transitions, comparable to those described by Shan et al. for PNIPAM-gold core-shell nanoparticles.

Nanoparticles in the size range 1–20 nm in diameter require polymer brushes of similar thickness (molecular weight and grafting density) for colloidal stability in aqueous dispersions. However, in this size range the curvature of the brush comprising the shell changes dramatically with core diameter. In this work, we investigate for the first time if the core diameter (nanoparticle curvature) has a strong influence on the CST of the shell. While it previously has been suggested that the internal morphology of the curved polymer shell influences the polymer solvation transitions [8,9,13,14], we unambiguously identify, for the first time, the different shell transitions. We also define which transition of the shell determines the onset of core-shell nanoparticle aggregation by comparing the CST of the polymer shells investigated using DSC to the colloidal aggregation transitions observed using dynamic light scattering (DLS). To facilitate this comparison, we refer to the measurement of the solvation transitions of the polymer itself using DSC as corresponding to the CST of the polymer. Measurements of particle aggregation using DLS are instead referred to as the critical flocculation (CFT) of the core-shell nanoparticle dispersion. To obtain data for nanoparticles with closely controlled variation of the curvature of the polymer brush, four different iron oxide core sizes with very narrow size distributions were synthesized: 5.1 ± 0.6, 7.4 ± 0.5, 10.2 ± 0.7 and 20.9 ± 1.3 nm. PiPOx, which exhibits a lower critical solution temperature in aqueous dispersions, was used to graft a dense polymer brush shell onto these cores. The MW of the PiPOx grafted to the particle surfaces was kept constant by using polymer from the same synthesis. The grafting densities of the polymer brushes grafted to each core size were carefully controlled to be similar using methods for ligand replacement, irreversible grafting and purification developed previously [8,13,15,16,17]. 

## 2. Materials and Methods 

### 2.1. Materials

Dopamine hydrochloride, sodium nitrite, sulfuric acid (98%), isobutyronitrile, ethanolamine, zinc acetate dihydrate (ZnAc_2_), anhydrous *N*,*N*-dimethylacedamide (DMA), methyl-*p*-tosylate (MeTs), succinic anhydride, 4-(dimethylamino)pyridine (DMAP), *N*,*N*-diisopropylethylamine (DIPEA) and anhydrous *N*,*N*-dimethylformamide (DMF) were purchased from Merk KGaA (Darmstadt, Germany) and were used as received unless noted otherwise. 1-[(1-(cyano-2-ethoxy-2-oxoethylidenaminooxy)-dimethylaminomorpholino)]-uronium-hexafluorphosphat (COMU), ethanol, dichloromethane (DCM), diethyl ether (Et_2_O), hexane and dry chloroform were purchased from Carl Roth (Karlsruhe, Germany) and were used as received. Dialysis tubes with different molecular weight cut-off (3.5 kDa: regenerated cellulose and 1000 kDa: cellulose ester, Spectra/Pro^®^, Float-A-Lyzer^®^ G2) were purchased from Carl Roth (Karlsruhe, Germany). 

**Nanoparticle synthesis:** Particles with sizes 5.1, 7.4 and 10.2 nm were synthesized using the precursor iron(0)pentacarbonyl. Highly monodisperse iron oxide nanoparticles were synthesized as described previously in detail [18,19]. Briefly, oleic acid coated iron oxide particles were prepared by thermal decomposition of iron(0)pentacarbonyl in presence of the surfactant oleic acid in the high boiling solvent dioctyl ether. Nanoparticles with size 20.9 nm were synthesized using the precursor iron oleate, as reported previously in detail [20,21]. Nanoparticles were obtained by thermal decomposition of iron oleate in the presence of oleic acid as surfactant in dioctyl ether. 

**Synthesis of 6-nitrodopamine (NDA):** 2.5 g (13 mmol) dopamine hydrochloride and 3.18 g (46 mmol) were dissolved in 75 mL degassed water. The solution was cooled with ice to 0 °C. 12.5 mL of 20% sulfuric acid was slowly added. After the addition of sulfuric acid, the reaction was warmed to room temperature and was stirred for 16 h. The product precipitated and was collected by filtration. It was resuspended in ethanol at 40 °C and precipitated with Et_2_O. Yield: 1.9 g (50%). ^1^H-NMR (300 MHz; DMSO-d_6_) δH: 7.45 (1H, s, Ar–H), 6.78 (1H, s, Ar–H), 3.04 (4H, m, CH_2_CH_2_–NH_3_).

**Synthesis of 2-isopropyl-2-oxazoline:** The monomer was synthesized using a modified Witte−Seeliger cyclocondensation [22,23]. 45 mL (0.5 mol) of isobutyronitrile was mixed with 36 mL (0.6 mol) of ethanolamine in the presence of 2.2 g (0.01 mol) zinc acetate dihydrate as catalyst. The reaction solution was kept at 130 °C for 24 h. The product was purified by extraction; therefore, it was dissolved in DCM and was extracted against water until the pH of the aqueous phase was neutral. The product was distilled over calcium hydride to yield an anhydrous product. 

**Polymerization of 2-isopropyl-2-oxazoline:** A 25 vol % solution of 2-isopropyl-2-oxazoline (10 mL) in anhydrous *N*,*N*-dimethylacetamide (DMA) (30 mL) was mixed with 72.4 µL of MeTs and stirred at 100 °C for to 24 h in a flame dried round bottom flask. The polymerization was quenched with 0.1 molar aqueous NaOH solution (1 mL). The polymer was isolated by precipitation with Et_2_O and hexane (ratio 1:1). The MW was determined by gel permeation chromatography (GPC) with polystyrene standards, resulting in a molecular weight of 26.3 kg mol^−1^ with a PDI of 1.05. Yield 9.4 g.

**Carboxy terminated polymer (PiPOx-COOH):** A previously published protocol for the functionalization of the polymer was followed [8,10]. The polymer (PiPOx, 9.4 g, 0.36 mmol) was dissolved in 80 mL dry chloroform. Thereafter, 400 mg (4.0 mmol) succinic anhydride and 161 mg (1.3 mmol) DMAP were added. The reaction mixture was refluxed for 24 h. The product was purified by extraction against water (twice 100 mL) and dried over Na_2_SO_4_. Yield: 9.4 g.

**Nitrocatechol-terminated polymer (PiPOx-NDA):** Carboxy-terminated PiPOx (0.36 mmol, 9.4 g) dissolved in 50 mL anhydrous DMF in inert atmosphere. Thereafter, 395 mg (0.92 mmol) COMU and 500 µL DIPEA were added and stirred for 15 min. NDA (346 mg, 1.2 mmol) was added as a solution in anhydrous DMF. The reaction solution was stirred for 48 h. The polymer was precipitated into 1:1 mixture of Et_2_O and hexane. The product was further purified by dialysis (cut off: 3.5 kDa) for 5 days. Degree of functionalization 66%. Overall yield: 4.7 g, 50% ^1^H-NMR for PiPOx-NDA δH (300 MHz; CD_3_OD) 7.53 (1H, s, Ar–H), 6.71 (1H, s, Ar–H), 4.27 (2H, CH_2_OCO–), 3.53 (4nH, –N–CH_2_CH_2_–PiPOx), 3.03–2.77 (1nH, CH(CH_3_)_2_, PiPOx), 1.10 (6nH, CH(CH_3_)_2_, PiPOx).

**Ligand exchange on SPION (“grafting to” of PiPOx):** The reaction of the particle batch with the core size of 5.1 nm is presented as a representative sample. Other particles were functionalized according to the same protocol with adjusted molar ratios to take into account the different specific surface areas of the samples. 269 mg wet oleic acid-coated SPION (inorganic fraction 25%) were dissolved in 1 mL toluene. 1.2 g nitrocatechol-terminated PiPOx were dissolved in 12 mL DMF. Both solutions were mixed and reacted under ultrasonication for 48 h. The temperature was kept below 30 °C. The particles were precipitated with Et_2_O/hexane (ratio 1:1) and dialyzed against water (cut off 1000 kDa) for 7 days. Yield: 83 mg. 

### 2.2. Methods

**^1^H-NMR** measurements were recorded on a BRUKER AV III 300 MHz spectrometer (Brucker Austria GmbH, Vienna, Austria). As reference residual protonated solvents CDCl_3_: 7.26 ppm, DMSO-d_6_: 2.50 ppm and CD_3_OD: 3.31 ppm were used. The chemical shift is given in ppm.

**Gel permeation chromatography (GPC)** was performed on a Malvern Viscotek GPCmax (Malvern Instruments Ltd., Worcestershire, UK) system to determine relative MW. The setup holds three MZ Gel SDPlus columns (a pre-column followed by two columns with separation ranges of 10–2000 kDa and 1–40 kDa, respectively). For detection, a Knauer Smartline RI Detector 2300 was used. As eluent DMF containing 0.05 M LiBr was applied with a flow rate of 0.5 mL min^−1^. 50 µL (concentration: 3 g L^−1^) of each sample was injected and measured at 60 °C. For the analysis, OminSEC 5.12 was used. The system was calibrated with polystyrene standards of 1.5–651 kg mol^−1^.

**Transmission electron micrographs (TEM)** were recorded on a FEI Tecnai G2 (FEI Europe B.V., Vienna, Austria), with 160 kV acceleration voltage on carbon-coated grids. The size distribution of each nanoparticle batch was based on the analysis of >300 NP imaged by TEM and analysed using the freeware Pebbles [24].

**Thermal gravimetric analysis (TGA)** was recorded on a Mettler Toledo TGA/DSC (Mettler Toledo GmbH, Vienna, Austria) (80 mL min^−1^ synthetic air, 20 mL min^−1^ nitrogen). The measurement was carried out in a temperature range from 25 to 650 °C with a heating rate of 10 K min^−1^. The grafting density (σ) was calculated from the TGA, GPC and TEM results using the following formula:

(1) $$\sigma =\frac{{\left(\%\;w/w\right)}_{shell}\;\rho_{iron\;oxide\;}V_{core}\;N_{A}}{{\left(\%\;w/w\right)}_{core}\;M_{polymer\;}A_{core}}\;$$

where ${\left(\%\;w/w\right)}_{shell}$ is percentage of mass loss in TGA for the organic fraction corresponding to the polymer grafted to the iron oxide core, $N_{A}$ is the Avogadro constant, $\rho_{iron\;oxide}$ is the density of iron oxide, $V_{core}$ is the volume and $A_{core}$ is the area of the iron oxide core calculated from the diameter of the cores measured by TEM, $M_{polymer}$ is the MW of polymer and ${\left(\%\;w/w\right)}_{core}$ is the residual mass percentage of the inorganic fraction in TGA.

**DLS measurements** ($D_{H}$, CFT and temperature cycling experiments) were performed in Milli-Q water on a Malvern Zetasizer Nano-ZS (Malvern Instruments Ltd., Worcestershire, UK). Mean values and standard errors of the number weighted diameter were calculated from three measurements for each temperature step. The CONTIN algorithm was used to extract size distributions from each measurement of the correlation curve. Temperature-cycled experiments were performed in the temperature range from 25 to 50 °C with a step-size of 1 °C. After changing the temperature, the sample was equilibrated for 2 min and then measured 3 times. Each reported value is an average of 11 runs. The CFT of the particle dispersions were determined by the onset of the increase of the hydrodynamic radius versus temperature. 

**Microdifferential scanning calorimetry** measurements on nanoparticle dispersions and polymer solutions in Milli-Q water were performed using a Malvern MicroCal PEAQ-DSC (Malvern Instruments Ltd., Worcestershire, UK) automated system. All samples were measured in the temperature range from 20 to 100 °C with a heating rate of 1 K min^−1^. Data processing was performed using the MicroCal PEAQ-DSC software version 1.22. The CSTs of the various transitions identified within the shell were determined as the peak maximum of the heat capacity versus temperature of the respective transition using DSC. The total enthalpy and the transition enthalpies per monomer unit were calculated from DSC heating curves using data from TGA and GPC. TGA gives the polymer weight fraction of the PiPOx-grafted SPION, which can be used to calculate the molar amount of PiPOx in the dispersed SPION sample used for DSC. The number average molecular weight of PiPOx was measured by GPC. Division by the monomer MW gives the degree of polymerization. The monomer transition enthalpy is then calculated using the following formula:

(2) $$\;\Delta H_{monomer\;unit}\left(\mathrm{kJ}\;{\mathrm{mol}}^{-1}\right)=\frac{Peak\;area\;\left(\mathrm{kJ}\right)}{n_{PiPOx}\left(\mathrm{mol}\right)\cdot DP}$$

**Statistical analysis** was carried out using DataLab (Version 3.530) (Epina GmbH, Pressbaum, Austria).

**Concentrations.** To make meaningful comparisons in terms of phase diagrams the concentrations of nanoparticle samples were chosen to have the same polymer molarity (nominally volume fraction). The nanoparticle samples have different core sizes and different number of grafted polymers per core (same grafting density but different surface area), which leads to different mass per particle and different organic: inorganic ratio. This means that different particle molarities and mass concentrations were used for different particle sizes to achieve the same polymer concentration. Throughout the manuscript, if nothing else is stated, we refer to the polymer concentration of each sample, which can be directly compared and used for creation of phase diagrams of the polymer solubility. Table 1 gives the conversion between the different measures of sample concentration. 

## 3. Results and Discussion

### 3.1. Preparation and Characterization of the Core-Shell Nanoparticles

**Figure 1:**
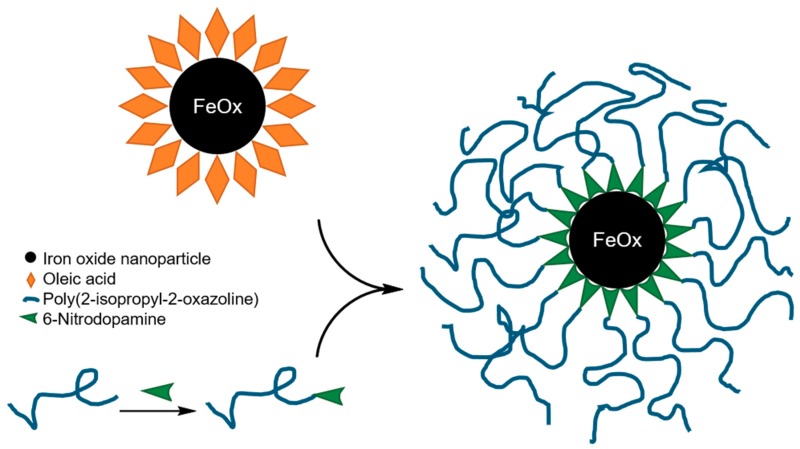
Preparation of the thermoresponsive core-shell iron oxide nanoparticles.

The “grafting to” method was used for the preparation of the thermoresponsive core-shell nanoparticles. The approach is schematically depicted in Figure 1 and follows our previously published work on synthesis of monodisperse polymer-grafted iron oxide nanoparticles [8,10,19]. The “grafting to” method has many advantages. First, the polymer can be fully characterized before the grafting is carried out to ensure it is monodisperse. Thus, differences in responsive properties between grafted polymer and free polymer coils can be compared. Second, it is easier to control the polymerization and uniform grafting to the nanoparticle core with the “grafting to” than with the “grafting from” method. A disadvantage of the “grafting to” method is that high grafting densities can be difficult to achieve when polymers with high MW are investigated. 

The anchor chemistry used to graft the polymer to the nanoparticle surface is of great importance [25]. This is particularly important if experiments involve extensive environmental changes such as large temperature and pH changes. An absolute requirement of the anchor for the ligand replacement is that the anchor has a higher affinity to the inorganic core than the ligand used for the synthesis of monodisperse cores has, i.e., higher than oleic acid for iron oxide nanoparticles. In our case, 6-nitrodopamine (NDA) was used as an anchor molecule. It binds with covalent character to ferric compounds [15,16,17]. NDA has been used to obtain both high grafting density and high stability of polymer brush coatings of iron oxide nanoparticles at high temperature [15,26]. We synthesized monodisperse core-shell nanoparticles with four different iron oxide core diameters grafted with PiPOx of the same molecular weight (26 kg mol^−1^) reported in Table 2. Details and references to the synthesis and purification are found in the Materials and Methods.

**Figure 2:**
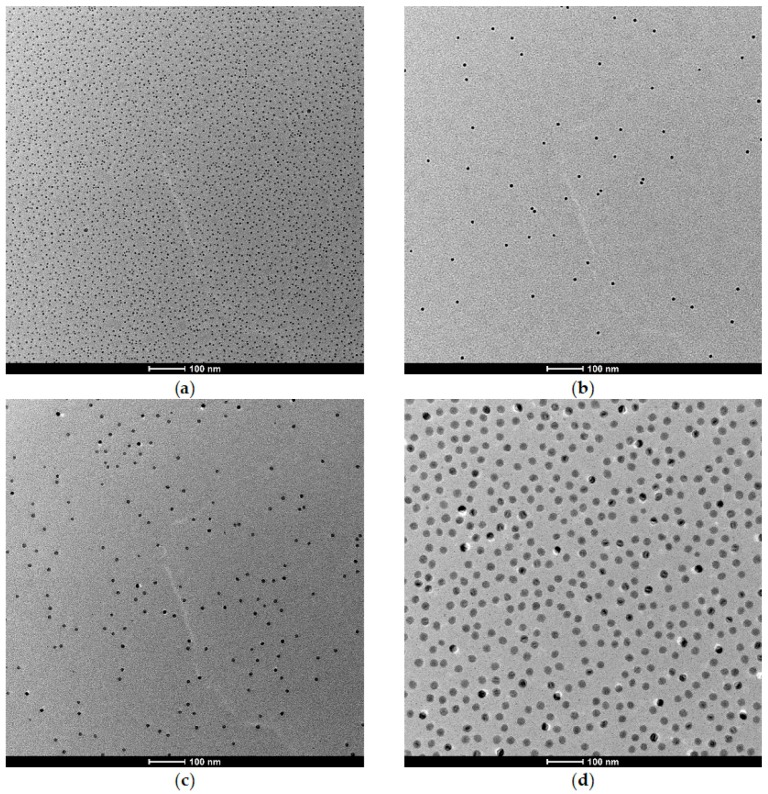
Transmission electron microscopy (TEM) micrographs of iron oxide nanoparticles grafted with poly(2-isopropyl-2-oxazoline) (26 kDa). The iron oxide core size was varied (**a**) 5.1 ± 0.6 nm; (**b**) 7.4 ± 0.5 nm; (**c**) 10.2 ± 0.7 nm; (**d**) 20.9 ± 1.3 nm. Scale bar corresponds to 100 nm.

Transmission electron microscopy (TEM) images of the PiPOx-grafted iron oxide nanoparticles are presented in Figure 2, with additional high-resolution images presented in Figure S1 of the Supporting Information. TEM generally shows only the cores due to the lack of electron density contrast of the polymer shell. However, a uniform distribution on the grid with well separated cores without aggregation can be observed for all samples. Figure S1e also shows a sample TEM of core-shell nanoparticles with a negatively stained background, which allows for indirect visualization of the dried shell on the grid. All particles show excellent colloidal stability and disperse spontaneously in water up to polymer concentrations >10 g L^−1^. The required high grafting density was determined by combining thermal gravimetric analysis (TGA) results (Figure S2) with the core size determined by TEM, as described in the Materials and Methods. The grafting densities and TGA results are provided in Table 2. Hydrodynamic diameters, $D_{H}$, of the nanoparticles were determined using dynamic light scattering (DLS) in Milli-Q water below the critical flocculation temperature. The average $D_{H}$ and standard errors are reported in Table 2. Corresponding particle size distributions for all measurements at temperatures below the CFT are found in the Supporting Information (Figure S3).

### 3.2. Temperature Dependence of Aggregation: Free Polymer versus Core-Shell Nanoparticles

The thermal responsiveness of all samples was studied using DLS and DSC. DLS measurements are used to determine the temperature at which the particles flocculate, the so-called critical flocculation temperature (CFT). The CFT describes the temperature of the onset of the global transition of the whole core-shell nanoparticle system, i.e., the flocculation or aggregation of the nanoparticles. The CFT is usually determined by measuring the loss of transmittance of the sample as aggregates are formed and scatter strongly in a standard UV/Vis spectrometer, so-called turbidity measurements. This approach only works well if the sample turns turbid due to large-scale inhomogeneities in the sample. This is not the case for our core-shell nanoparticle dispersions. However, DLS can be used to determine the CFT by tracking the size distribution of particles and particle aggregates as a function of temperature. The scattered light intensity measured by the DLS detector is strongly dependent on the size of particles and aggregates, as $I_{scattered}\propto D^{6}$. Monitoring the sudden increase in $I_{scattered}$ with increased temperature is thus an additional sensitive way to determine the particle aggregation temperature using DLS. This measurement technique is analogous to established turbidity measurement but more sensitive and does not require that the colloidal dispersion turns turbid.

**Figure 3:**
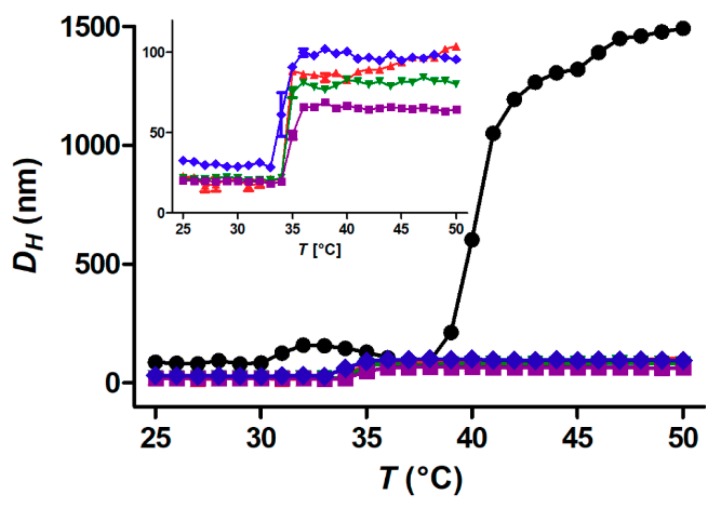
Dynamic light scattering (DLS) heating curves of 1 g L^−1^ samples: black: free polymer, red: FeOx-5, green: FeOx-7, violet: FeOx-10 and blue: FeOx-21. The inset shows the same data exclusively for the nanoparticle dispersions.

Table 3 and Figure 3 show the average hydrodynamic size ($D_{H}$) of all nanoparticles plus the free polymer samples measured by DLS upon heating at a polymer concentration of 1 g L^−1^ in Milli-Q water. The individual heating and cooling curves recorded by DLS for all investigated polymer concentrations are found in the Supporting Information (Figures S4–S8). The observed changes in $D_{H}$ all correlated with similar rapid changes in scattered intensity (DLS count rate). Traditional turbidity measurements were not performed to monitor the transitions since none of the samples was turbid.

Note that the polymer concentration is the same in all samples. This means that the nanoparticle concentration (volume fraction) varies according to Table 1. The first thing to observe is that the CFT is shifted to lower temperature after the grafting of the polymer to the nanoparticle core, as previously described [8,9,13]. The CST of the free polymer is 38 °C, while the CFT of the core-shell nanoparticles is ~34 °C. The CFT shows only minor dependence on particle core size. The largest 21 nm core particles have a CFT of ~33 °C. A second observation is that the core-shell nanoparticle aggregates are one order of magnitude smaller than those of the free polymer despite the much larger nanoparticle size. Also, the core-shell nanoparticles show almost perfectly reversible aggregation and deaggregation without hysteresis. Only the smallest 5.1 nm cores display a slight hysteresis. In contrast, the free polymer aggregates only display partial deaggregation upon cooling over the minute time-frame of the experiment (*cf*. Figures S4–S8).

**Figure 4:**
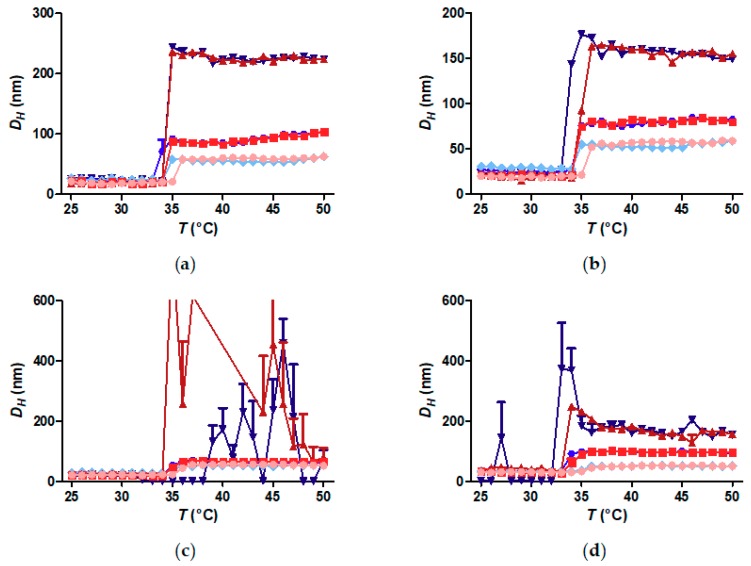
Temperature-cycled average hydrodynamic size measured by dynamic light scattering of PiPOx (26 kDa)-grafted iron oxide nanoparticles at different concentrations. Heating curves are depicted in red colours, while cooling curves are depicted in blue colours. Light red and light blue: polymer concentration: 0.1 g L^−1^, red and blue: polymer concentration: 1 g L^−1^ and dark red and dark blue: polymer concentration: 10 g L^−1^. (**a**) FeOx-5; (**b**) FeOx-7; (**c**) FeOx-10; (**d**) FeOx-21.

The concentration dependence of the critical solution temperature of free polymer coils is well-known and understood [27]. It was also recently investigated for core-shell nanoparticles, where ad hoc comparison of core-shell nanoparticle concentrations revealed a dependence of both the CFT and the aggregate size on concentration [8,13]. Figure 4 directly compares the CFT and aggregate size of core-shell nanoparticles with different core sizes as function of the polymer concentration. Figure 4 shows $D_{H}$ for the heating (red) and cooling (blue) curves measured by DLS at polymer concentrations 0.1, 1.0 and 10 g L^−1^ for each core size. Clearly, the concentration has only a minor impact on the CFT of the nanoparticle dispersions. The difference between highest and the lowest CFT is not more than ~1 °C. The size of the nanoparticle aggregates above the CFT at a given concentration is stable and depends on the concentration. Higher concentration leads to larger aggregates, as previously reported [8,13]. However, the aggregate size is not dependent on nanoparticle core size. This is interesting in view of a previous study in which we showed a strong dependence on nanoparticle shell size (grafted polymer molecular weight) [8]. A larger core leads to stronger van der Waals attraction between the particles. This interaction is not concentration dependent. However, changing the particle size by changing the molecular weight of the grafted polymer changes the volume fraction of polymer in the sample, which favours larger aggregates to be formed. Additionally, free polymers form larger aggregates (cf. Figure 3). Grafting higher MW polymer to a core makes the particle more like free polymer, everything else being equal. These considerations are likely to explain the contrasting results for these two studies in which the overall core-shell particle size is varied.

### 3.3. Critical Solution Temperature and Transition Enthalpies of Polymer Grafted to Nanoparticles

A distinction should be made between the CFT and the local change in solubility of the polymer chains. The latter is denoted the critical solution temperature (CST). The CST can be measured most directly by differential scanning calorimetry (DSC). It measures the heat required to keep the sample at the same temperature as the pure continuous phase during a temperature scan. Thus, each phase transition can be recorded, as the temperature is scanned, by the change in heat capacity as bonds are broken and formed. The enthalpy of the transitions can be determined by integrating this data. The solvation of a polymer in water is thus measured by DSC through the heat required to form or break hydrogen bonds between water and polymer. For a composite colloidal system, the change of the polymer solvation, referred to by the CST at which it occurs, can be a very broad transition. The CST therefore does not have to correspond exactly to the temperature at which the particles flocculate, that is the CFT. We will henceforth refer to the solubility transitions of the polymer as CST transitions and to the colloidal aggregation of the core-shell nanoparticle dispersions or free polymer solutions as CFT transitions.

**Figure 5:**
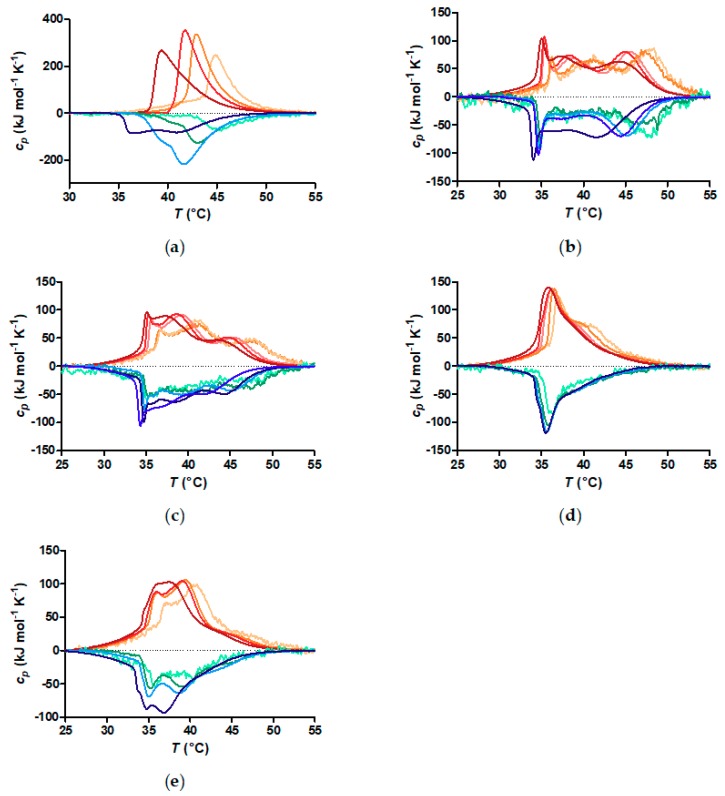
Dynamic scanning calorimetry (DSC) curves of free polymer and core-shell nanoparticles, orange-red: heating curves and green-blue: cooling curves. (**a**) Free polymer: light orange and light green: 0.1 g L^−1^, orange and green: 0.5 g L^−1^, red and blue: 1 g L^−1^and dark red and dark blue: 10 g L^−1^; (**b**) FeOx-5: light orange and light green: polymer concentration: 0.06 g L^−1^, orange and green: polymer concentration: 0.1 g L^−1^, light red and light blue polymer concentration: 0.5 g L^−1^, red and blue: polymer concentration: 1 g L^−1^ and dark red and dark blue: polymer concentration: 10 g L^−1^; (**c**) FeOx-7: light orange and light green: polymer concentration: 0.1 g L^−1^, orange and green: polymer concentration: 0.112 g L^−1^, light red and light blue polymer concentration: 0.5 g L^−1^, red and blue: polymer concentration: 1 g L^−1^ and dark red and dark blue: polymer concentration: 10 g L^−1^; (**d**) FeOx−10: light orange and light green: polymer concentration: 0.1 g L^−1^, orange and green: polymer concentration: 0.28 g L^−1^, light red and light blue: polymer concentration: 0.5 g L^−1^, red and blue: polymer concentration: 1 g L^−1^ and dark red and dark blue: polymer concentration: 10 g L^−1^; (**e**) FeOx-21: light orange and light green: polymer concentration: 0.1 g L^−1^, orange and green: polymer concentration: 0.5 g L^−1^, red and blue: polymer concentration:1 g L^−1^ and dark red and dark blue: polymer concentration: 10 g L^−1^.

A core-shell nanoparticle has the polymer chains densely end-grafted to the core. This is expected to change their CST due to the change in local segment concentration, which imposes chain conformations different to that of the free coil. This is seen already from the decrease of the CFT after grafting of the polymer to the iron oxide cores. In Figure 5, the heating (red) and cooling (blue) curves for free polymer and core-shell nanoparticles measured by DSC are shown. The change in heat capacity ($c_{p}$) with temperature is not sensitive to the colloidal aggregation, but only to the breaking and formation of hydrogen bonds between polymer and water. The free polymer heating curves show a single peak with some hysteresis during cooling. Two peaks can be identified during cooling. One of the peaks appear at lower temperature than the heating transition. As expected, the CST of the free polymer decreases as the concentration is increased, in good agreement with the CST measured by DLS. The polymer grafted to nanoparticles shows far more complex behaviour. Multiple peaks are identifiable in both heating and cooling curves. None of them is directly identifiable with the transition observed of the free polymer. Interestingly, the relative intensity of the different transition peaks changes with particle core size. The transition peaks at higher temperature decrease as the core size increases. The lowest temperature transition peak is essentially found at the same temperature in all samples. The higher temperature peaks also appear to stay at the same temperature as the size is increased, although it is difficult to tell by eye as their intensity decreases with increasing core size. 

The DSC data was used to calculate the enthalpy of the total transitions for each core size and concentration, both for heating and for cooling. The data is summarized in Table 4. Two things can be observed: (i) The transition enthalpy per monomer tends to be higher the less dense the polymer shell is, i.e., the higher the curvature of the shell is since a higher curvature yields a higher free volume per grafted chain. The highest transition enthalpy per monomer is observed for the free polymer coils and the lowest for the largest cores that have a more uniform segment density of the brush. (ii) The transition enthalpy per monomer is higher during heating than during cooling, i.e., not all polymer-water hydrogen bonds that were broken during dehydration are reformed upon cooling and rehydration.

### 3.4. Is the Aggregation of the Thermoresponsive Core-Shell Nanoparticles Fully Reversible?

Both the DLS and the DSC data described above, indicate that there is a difference between the heating and cooling parts of the cycle. The DSC results indicate that the PiPOx does not fully rehydrate upon cooling. The enthalpy change during cooling and rehydration is substantially smaller than that for heating and dehydration. This is observed for the free polymer as well as for the grafted polymer shells. Hoogenboom and co-workers have previously described that the remixing of free PiPOx coils is a slower process compared to the phase separation [28]. The reason for this could be that parts of the polymer undergo a conformation change from mixed gauche and trans to all trans [29]. This conformation change induces crystallization, which is not spontaneously reversible. Furthermore, it can be assumed that the dehydration is faster than the rehydration given that the diffusion of water out of the brush is faster than polymer chain rearrangement to allow water to diffuse into the brush.

A cursory look at the DLS data seems to indicate that the nanoparticle aggregation in contrast to the polymer desolvation is fully reversible within a short time scale. However, a closer analysis of the data shows that a fraction of the nanoparticles remains in small clusters. Table 5 shows $D_{H}$ before and after the thermal cycle for the samples with different core size at a polymer concentration of 1 g L^−1^. $D_{H}$ values in the temperature range from 25 °C to $T_{onset}-1$ were used. The differences are small, but a Wilcoxon Test for paired differences was carried out to demonstrate that the $D_{H}$ is not equal before and after the thermal cycle at a significance value of 95%. The larger average hydrodynamic size after redispersion implies that some particles remain in small clusters. Lower particle concentrations trend toward lower rehydration. This is roughly observed also from DSC (cf. Table 4), although a few data points lie outside the trend. 

### 3.5. Does the Curvature Influence on Internal Brush Structure Affect the CST of the Polymer Shell?

On flat surfaces, the polymer segment volume fraction of grafted polymer chains is near constant throughout the polymer brush layer. The major influence on the polymer segment volume fraction is the grafting density of the polymer on the surface, the excluded volume (hydration) and flexibility of the monomer segments. If the surface is a nanoparticle, the polymer volume fraction exhibits a radial gradient, with a functional dependence of the polymer volume fraction that varies with distance from the highly curved surface. Close to the surface there is a densely packed shell, which is similar to brushes grafted to flat surfaces. It can have a near constant polymer segment volume fraction if the grafting density is high, in analogy to star polymers [30,31,32]. Outside this region, a spherical brush segment density profile is expected, which follows a $r^{-4/3}$ decrease with distance from the surface. If the polymer molecular weight is comparable to the core diameter or larger, then the segment density in the outermost part of the grafted polymer shell will reach a concentration similar to that of the free coil. Thereby, the polymer conformation in the outer part of a highly curved polymer brush is comparable to that of chains grafted in the mushroom regime on planar surfaces. Thus, three density or polymer volume fraction regimes can theoretically be expected within a spherical brush shell densely grafted on a highly curved nanoparticle core. As the CST depends on where we are in the polymer-solvent phase diagram, that is on the local polymer segment volume fraction, this should theoretically lead to multiple or broadened transitions or CSTs for highly curved nanoparticles. The size of the free 26 kDa PiPOx we graft is similar in dimension to the nanoparticle cores. However, we significantly vary the curvature by changing the size of the particle core from 5 to 21 nm in core diameter. Thereby, we vary the relative fraction of the polymer shell that is expected to be in each of the concentration regimes for the different core sizes.

Although in practice these theoretically discernible regimes [31,33] should merge into each other. It is clear from Figure 5 that the DSC data indicates three transitions in each temperature scan. It is tempting to correlate the three regimes within the polymer segment profiles of the spherical brush with the three transitions observed by DSC. The innermost part of the shell can be assigned to the peak at the lowest temperature in the DSC scans. Supporting this interpretation, the peak at the lowest temperature is found close to the same temperature regardless of curvature (core size), i.e., for all the investigated particles. This part of the shell should have almost constant polymer volume fraction. Here, the polymer density depends only on grafting density, and therefore is expected to be similar for all particles. Furthermore, it was previously observed that the polymer block closest to the core showed the lowest CST by DSC for NDA-PiPOx-*b*-PEtOx and NDA-PEtOx-*b*-PiPOx block copolymer grafted to inorganic cores respectively [14]. The other two peaks occur at similar temperatures for particles of different size and curvature. It is obvious from Figure 5 that their fraction of the total enthalpy of the transition as well as their dependence on concentration (discussed in more detail below) are different. Since a higher polymer volume fraction should lead to a lower CST, it is logical to attribute the intermediate transition peak to the part of the shell with the brush density decreasing as $r^{-4/3}$. The transition peak at the highest temperature is the attributed to the mushroom- or free coil-like outermost part of the polymer shell. The two peaks at higher temperature have a strong overlap that changes with a change in particle concentration.

**Figure 6:**
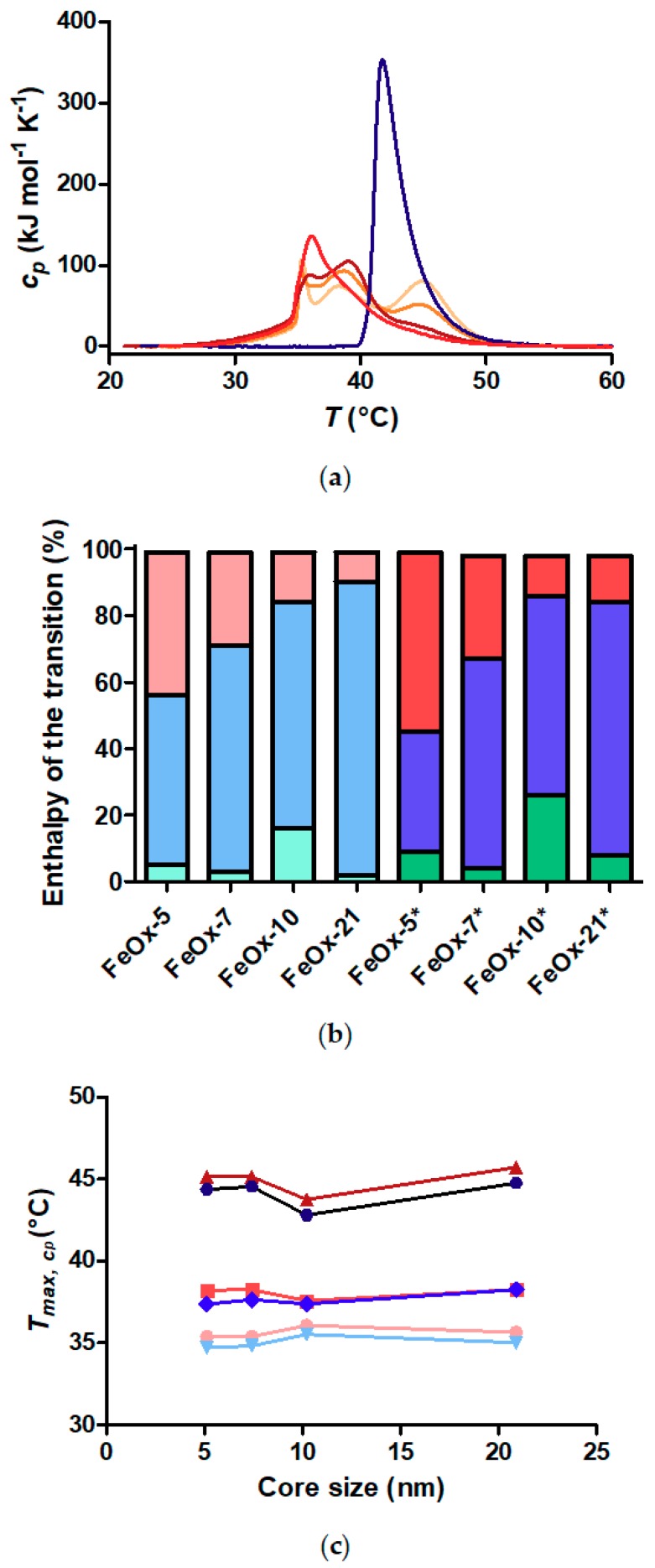
DSC transition peak analysis. (**a**) Overlay of DSC endotherms of free polymer (dark blue), FeOx-5 (light orange), FeOx-7 (orange), FeOx-10 (red) and FeOx-21 (dark red) at a polymer concentration of 1 g L^−1^; (**b**) Fraction of the total transition enthalpy of each fitted transition peak for core-shell nanoparticles. All DSC curves were fitted with three Gaussian curves. The enthalpy of each fitted Gaussian was normalized by the total enthalpy of the transition to obtain the plotted fractions. Each bar presents the contribution to the total peak area at a polymer concentration of 1 g L^−1^. Contributions of the lowest (green), intermediate (blue) and highest (red) temperature transitions were assigned to the inner, intermediate and outer shell, respectively. Heating curves are left and cooling curves are right, with the latter marked by *; (**c**) Temperature of maximum $c_{p}$ ($T_{max,c_{p}}$) of each deconvoluted transition peak at a polymer concentration of 1 g L^−1^.

It is obvious that the core size has major impact on the outer shell from the direct comparison of the DSC data for nanoparticles at the same concentration in Figure 6a. Free polymer has a single transition peak, while the core-shell nanoparticles display three peaks. The transition peak at the highest temperature, which corresponds to the outermost part of the shell, is reduced as the core size is increased and the curvature decreased. Correspondingly, the peak at intermediate temperature seems to increase as the core size is increased, as more of the shell is in the star polymer-like than the mushroom-like regime. 

To make a quantitative comparison of the features observed in the DSC curves, all DSC curves were fitted with three Gaussian curves. Gaussian peak fits are unlikely to lead to perfect deconvolution of the actual transitions, as it has no basis in a physical model of the brush. However, it aids us to better distinguish and quantify the enthalpy of each transition and the temperature at the maximum $c_{p}$ of each transition. Supporting this approach is also that the Gaussian fits reproduce the DSC curves with a very small error, as shown for all peak fits in Figures S9–S13. In Figure 6b, we use these fits to visualize the fraction of the enthalpy of the total transition attributed to each region of the shell. The fraction of the shell displaying the mushroom-like character of the outermost shell decreases on average as the core size is increased. The transition enthalpy of the intermediate part of the shell correspondingly increases as a fraction of the total enthalpy of the transition. Interestingly, these changes take place without a significant change in the temperature at which the respective transition takes place, according to the positions of the best-fit peaks, as shown in Figure 6c. Finally, hysteresis is observed for the shell transitions. The transitions occur at slightly lower temperature upon cooling. Furthermore, it seems the transition of the intermediate part of the polymer brush shell contributes a smaller fraction of the enthalpy of the total transition for the cooling curve than for the heating curve. We will discuss the latter observation in more depth below using data on the concentration dependence of the transitions.

### 3.6. Does Nanoparticle Concentration Influence Brush CST and Thermally Induced Aggregation?

From previous reports it is known, that the thermal aggregation of PiPOx is dependent on concentration [28,34]. At low concentrations, a higher CST is observed compared with high concentrations close to the lowest critical solution concentration (LCST). As described in the section on DLS this can be observed for the aggregation of PiPOx. It is confirmed by the DSC measurements at different concentrations depicted in Figure 5a. These show that the maxima of the DSC curves shift to lower values with increasing concentration. The peak maximum at the lowest concentration of 0.1 g L^−1^ is found at a temperature of $T_{max}=$ 45 °C, while $T_{max}$ is observed at 39 °C at a concentration of 10 g L^−1^. This effect is even more pronounced studying the cooling instead of the heating DSC curves. At the lowest concentration of 0.1 g L^−1^, the peak maximum temperatures for heating and cooling are identical. In contrast, $T_{max}$ is shifted by −3 °C for the cooling curve compared to the heating curve at the highest concentration. 

As described above (Figure 4), we hardly observe a concentration dependence of the CFT for nanoparticles grafted with PiPOx. However, the DSC measurements of the polymer shell CSTs show concentration dependence. In Figure 5 we observe that if the concentration is increased, the two higher temperature transitions shift closer to the lowest temperature peak. The lowest temperature peak remains at the same temperature. At the highest concentration of FeOx-21 (10 g L^−1^ polymer), the low and intermediate temperature transition peaks have merged into one broad peak. This result is similar to an observation made by Shan et al. [11]. At low concentrations, they found two easily distinguishable solvation transitions within the shell of PNIPAM-grafted gold nanoparticles with a diameter of 1.3 nm. However, at higher nanoparticle concentrations the two transitions merged to apparently one transition [11]. This finding was explained by that the relative difference in local volume fraction between the two shells decreases when the overall volume fraction of the polymer in the bulk increases with the core-shell nanoparticle concentration. We conclude that this interpretation is supported by our more detailed results on the relationship between curvature-dependent internal brush structure and solvation transitions of the polymer shell. As the nanoparticle and thereby polymer volume fraction in the sample is increased, the local volume fraction and therefore chemical potential changes much more for the outer part of the shell. This is a result of that the relative difference to the bulk of the polymer segment concentration is lower for the outer than for the inner shell of a curved polymer brush.

**Figure 7:**
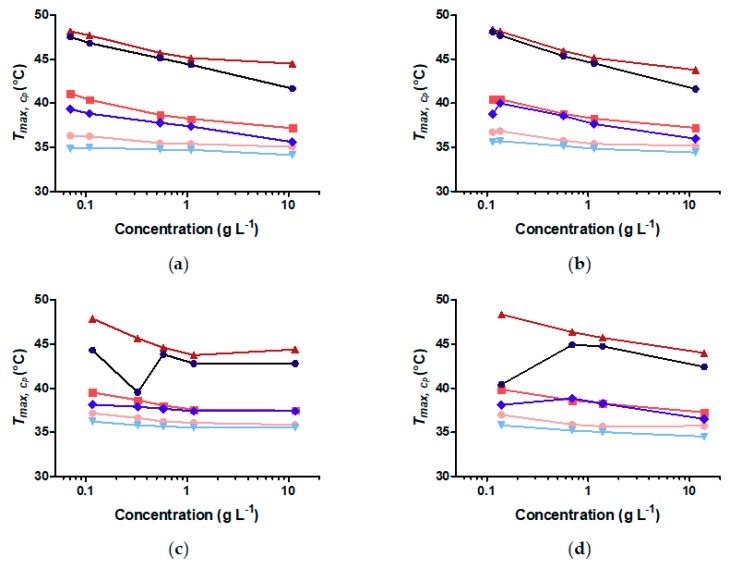
Temperature of maximum $c_{p}$ ($T_{max,c_{p}}$ ) of each deconvoluted transition peak plotted vs. nanoparticle concentration. The red curves represent the heating curves and the blue curves the cooling curves. The light red and light blue curves represent the inner shell, red and blue curves represent the intermediate shell and dark red and dark blue curves the outer shell. (**a**) FeOx-5; (**b**) FeOx-7; (**c**) FeOx-10; (**d**) FeOx-21.

The temperature at the maximum $c_{p}$ of each fitted peak, $T_{max,c_{p}}$, is plotted in Figure 7 for all particles as function of concentration. $T_{max,c_{p}}$ weakly depends on particle curvature. The lowest temperature peak corresponding to the innermost part of the shell hardly changes with particle concentration. The change in $T_{max,c_{p}}$ is less than 1.6 °C between the highest and the lowest concentration (0.1 and 10 g L^−1^ of the polymer concentration) for all different core sizes. However, Figure 5b–e and Figure 7 show that $T_{max,c_{p}}$ of the other two peaks is much more influenced by particle concentration. This reduces the difference in temperature between the positions of the peak corresponding to the inner part of the shell and those of the outer parts of the shell.

For the FeOx-21 sample at the highest concentration (10 g L^−1^ polymer), the first two maxima of the fitted Gaussian curves show only a small difference in $T_{max,c_{p}}$ of ~1.5 °C. The transitions of the two outermost parts of the shell increasingly overlap, making them more difficult to deconvolute. Increased hysteresis during the complete heating-cooling cycle is also observed (*cf*. Figure 5 and Figure 7). Most samples indicate the cooling transitions shifted to increasingly lower temperature with increasing particle concentration. This hysteresis can also be observed in DLS as shown in Figures S7 and S8.

**Figure 8:**
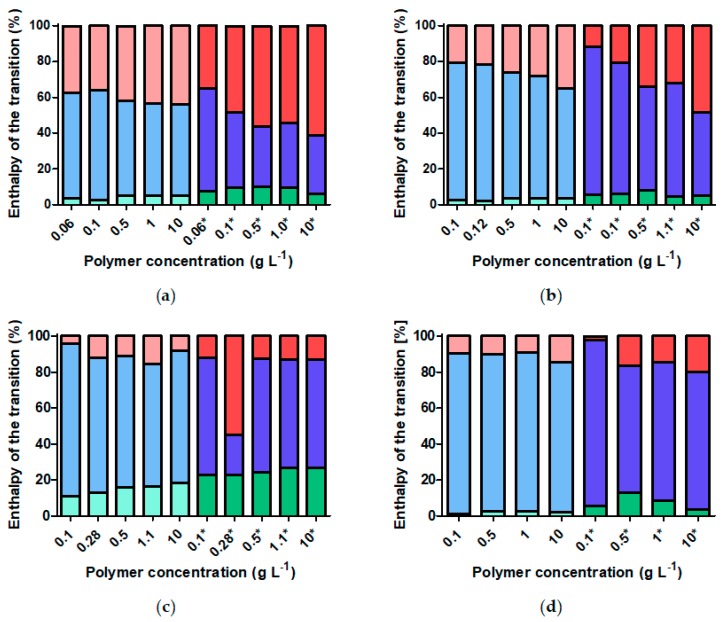
Fraction of the total transition enthalpy of each fitted transition peak for core-shell nanoparticles. All DSC curves were fitted with three Gaussian curves. The enthalpy of each fitted Gaussian was normalized by the total enthalpy of the transition to obtain the plotted fractions. Contributions of the lowest (red), intermediate (blue) and highest (green) temperature transitions were assigned to the inner, intermediate and outer shell, respectively. The cooling curves are marked with *. (**a**) FeOx-5; (**b**) FeOx-7; (**c**) FeOx-10; (**d**) FeOx-21.

In Figure 8, the enthalpy of each transition identified in the polymer shell is plotted normalized to the enthalpy of the complete transition. From this data, we can observe that the fraction of the transition enthalpy corresponding to the intermediate peak decreases with increasing concentration of the particles. Interestingly, this is observed to occur through an increase in the fractions of the enthalpy of both the inner and outer shell transition peaks, although the increase in the contribution of the outer shell transition is larger. This rather unexpected result is possibly explained as an artefact of the deconvolution by fitting Gaussian peaks that are increasingly overlapping as the concentration is increased. This observation is even more prominent in the cooling curves than in the heating curves. The inner and especially the outer shell transitions are larger parts of the total transition at all concentrations for the cooling curves than for the heating curves. This result can be explained by the hysteresis yielding a higher fraction of polymer at high local volume fraction in the cooling curve.

Finally, it is interesting to compare the correlation of the CFT determined by DLS to the various CST transitions determined by DSC. The CFT of the nanoparticle dispersions hardly changes with curvature or concentration. While the total transitions measured by DSC greatly vary with curvature and concentration, the CST of the innermost shell stays remarkably constant for the investigated core-shell particle dispersions. The CFT and the CST of the inner shell also are almost identical at 33–35 °C. Thus, it seems like the transition of the inner part of the shell alone controls at which temperature these particles aggregate. This is unexpected since naively the transition of the inner part of the shell should have the least influence on the colloidal interactions between the nanoparticles. Additionally, Figure 8 shows that the inner part of the shell contributes only a small fraction of the total transition and therefore likely contains a minor part of the polymer in the shell. This observation holds true for both aggregation and deaggregation. Possibly, the complete dependence of the CFT on the transition of the inner part of the shell is limited to a certain range of polymer molecular weights, that is shell thicknesses. As the molecular weight is increased and the remaining shell remains hydrated as the inner part of the shell collapses at low temperature, it is conceivable that the remaining solvated parts of the shell are sufficiently dense and thick to prevent particle aggregation. In the present study, the molecular weight of the grafted polymer was kept constant. However, there are indications in other studies [8] that a higher polymer molecular weight in relation to the core size might modify the correlation between inner shell CST and dispersion CFT observed here.

## 4. Conclusions

We have, for the first time, presented a comprehensive study on the thermoresponsiveness of iron oxide core-shell nanoparticles grafted with poly(2-isopropyl-2-oxazoline) as function of core size. Four different and highly monodisperse spherical cores were used, with average diameters of 5.1, 7.4, 10.2 and 20.9 nm, respectively. All particles showed excellent dispersibility and colloidal stability in water at room temperature, using 26 kg mol^−1^ nitrodopamide-poly(2-isopropyl-2-oxazoline) densely grafted at ~1.2 chains nm^−1^. Using DLS, we determined that changing the core size had little impact on the CFT of the nanoparticles, but there is a major difference in CFT of 4 °C (at 1 g L^−1^ polymer) between polymer brush stabilized nanoparticles and the corresponding free polymer. Particle core size, did not seem to influence cluster size during aggregation.

Using DSC, we could clearly distinguish the effect of nanoparticle curvature leading to a polymer volume fraction changing radially within the brush and corresponding to three distinct CST transitions within the brush. These were attributed to three different regions of the highly curved polymer brush shell, which each has a different radial dependence for the polymer volume fraction. A detailed investigation led to the following important findings regarding the thermal response of the respective shell regions:

(i) The inner shell shows high and almost constant polymer volume fraction. Its CST is nearly independent on size and concentration. Interestingly and unexpectedly, the CST of the innermost part of the shell corresponds closely to the CFT of the nanoparticle dispersion. The innermost part of the brush shell can thus be tailored to achieve the desired aggregation temperature of the nanoparticle dispersion, while being largely insensitive to other experimental parameters.

(ii) The central part of the shell shows a $r^{-4/3}$ radial decay in polymer volume fraction. Its CST is not strongly dependent on core size, but the fraction of polymer transitioning at this CST is strongly dependent on core size. Increasing particle concentration decreases this CST.

(iii) The outermost shell has a mushroom-like conformation and exhibits the strongest dependence on core size and concentration. The contribution of the outer shell to the total enthalpy decreases with increasing core size, as the conformation of the brush becomes increasingly similar to that of a homogeneous planar polymer brush. While concentration affects both CST and enthalpy of the transition, core size has only a minor influence on the CST of the outermost part of the shell.

The higher the particle concentration is, the more similar the different parts of the shell become. This effect is strongest for the two outermost parts, for which the respective desolvation transitions increasingly overlap and shift toward the transition temperature of the innermost part of the polymer brush. Some of these effects are observed even more strongly in the cooling than in the heating traces. The DSC reveals a quite strong hysteresis of the resolvation and internal reorganization of the shell, which is only weakly observed in a low fraction of remaining aggregates of the nanoparticle dispersion.

This knowledge can be combined with previous studies showing that nanoparticle cluster size can be controlled by MW of the polymer in the brush [13] and nanoparticle concentration [8] to design thermoresponsive nanoparticles optimized for applications where transition temperature, cluster size and reversibility must be tuned independently.

## Figures and Tables

**Table 1:** Comparison of particle and polymer concentrations for different core-shell nanoparticle samples.

Sample	Core-shell Particle Concentration (g L^−1^)	Grafted Polymer Concentration (g L^−1^)	Particle Molarity (mol L^−1^)
FeOx-5	0.07	0.06	2.7 × 10^−8^
FeOx-5	0.1088	0.1	4.0 × 10^−9^
FeOx-5	0.54	0.5	2.0 × 10^−7^
FeOx-5	1.088	1.0	4.0 × 10^−7^
FeOx-5	10.88	10	4.0 × 10^−6^
FeOx-7	0.114	0.1	2.2 × 10^−8^
FeOx-7	0.135	1.12	2.7 × 10^−8^
FeOx-7	0.57	0.5	1.1 × 10^−7^
FeOx-7	1.14	1.0	2.2 × 10^−7^
FeOx-7	11.4	10	2.2 × 10^−6^
FeOx-10	0.116	0.1	9.3 × 10^−9^
FeOx-10	0.324	0.28	2.7 × 10^−9^
FeOx-10	0.58	0.5	4.7 × 10^−8^
FeOx-10	1.16	1.0	9.3 × 10^−8^
FeOx-10	11.6	10	9.3 × 10^−7^
FeOx-21	0.139	0.1	2.7 × 10^−9^
FeOx-21	0.695	0.5	1.3 × 10^−8^
FeOx-21	1.39	1.0	2.7 × 10^−8^
FeOx-21	13.9	10	2.7 × 10^−7^

**Table 2:** Characteristics of iron oxide nanoparticles grafted with poly(2-isopropyl-2-oxazoline) a molecular weight of 26 kg mol^−1^.

Sample	Core Diameter (nm)	TGA			$D_{H}\;(\mathrm{nm})$
		Weight Loss (wt %)	Residue (wt %)	σ	
FeOx-5	5.1 ± 0.6	91.9	8.1	1.2	19.7 ± 4.9
FeOx-7	7.4 ± 0.5	87.3	12.7	1.0	21.4 ± 1.1
FeOx-10	10.2 ± 0.7	86.1	13.9	1.3	21.6 ± 1.6
FeOx-21	20.9 ± 1.3	72.1	27.9	1.1	30.5 ± 1.9

**Table 3:** Overview of $D_{H}$ of core-shell nanoparticles with a polymer concentration of 1 g L^−1^ and their critical flocculation temperature (CFT) measured by DLS.

Sample	$D_{H,T\;<\;CST}\;(\mathrm{nm})$	$D_{H,T\;>\;CST}\;(\mathrm{nm})$	CFT (°C)
Free Polymer	108.7 ± 5.0	1340.9 ± 25.1	38
FeOx-5	19.7 ± 1.0	91.7 ± 1.0	34
FeOx-7	21.4 ± 0.7	80.9 ± 0.4	34
FeOx-10	20.0 ± 0.3	65.5 ± 0.3	34
FeOx-21	30.5 ± 0.4	97.7 ± 0.5	33

**Table 4:** Enthalpy per monomer of the polymer solvation transition measured by DSC.

Sample	Grafted Polymer Concentration (g L^−1^)	$\Delta H_{monomer,heating}$ $\left(\mathrm{kJ}\;{\mathrm{mol}}^{-1}\;K^{-1}\right)$	$\Delta H_{monomer,cooling}$ $\left(\mathrm{kJ}\;{\mathrm{mol}}^{-1}\;K^{-1}\right)$	$\frac{\Delta H_{cooling}}{\Delta H_{\;heating}}\;(\%)$
**FeOx-5**	0.06	3.85	−3.16	82
**FeOx-5**	0.1	4.13	−2.29	55
**FeOx-5**	0.5	3.86	−2.65	69
**FeOx-5**	1.0	3.97	−2.88	72
**FeOx-5**	10	4.05	−3.71	92
**FeOx-5 mean**		3.97 ± 0.11	−2.94 ± 0.48	74
**FeOx-7**	0.1	3.75	−2.71	72
**FeOx-7**	0.12	3.76	−2.89	77
**FeOx-7**	0.5	3.85	−2.68	69
**FeOx-7**	1.0	3.80	−3.33	88
**FeOx-7**	10	3.73	−3.26	88
**FeOx-7 mean**		3.78 ± 0.05	−2.97 ± 0.27	79
**FeOx-10**	0.1	3.47	−1.66	48
**FeOx-10**	0.28	3.40	−2.35	69
**FeOx-10**	0.5	3.30	−2.37	72
**FeOx-10**	1.0	3.33	−2.37	71
**FeOx-10**	10	3.53	−3.08	87
**FeOx-10 mean**		3.41 ± 0.09	−2.37 ± 0.45	69
**FeOx-21**	0.1	3.64	−1.78	49
**FeOx-21**	0.5	3.70	−2.00	54
**FeOx-21**	1.0	3.67	−2.55	69
**FeOx-21**	10	3.58	−3.23	90
**FeOx-21 mean**		3.65 ± 0.04	−2.39 ± 0.56	65
**Free polymer**	0.1	4.46	−1.75	39
**Free polymer**	0.5	4.76	−2.66	56
**Free polymer**	1	4.85	−5.06	104
**Free polymer**	10	4.81	−3.04	63
**Free polymer mean**		4.72 ± 0.15	−3.13 ± 1.21	66

**Table 5:** Average particle/cluster hydrodynamic diameter before and after thermally induced aggregation in the temperature range from 25 °C to $T_{onset}-1$, measured and calculated for dispersions with 1 g L^−1^ concentration of polymer. The $D_{H}$ before and after are different with a significance value of 95%.

Sample	$D_{H,before}\;$	$D_{H,after}\;$
FeOx-5	19.7 ± 1.0	23.4 ± 0.7
FeOx-7	21.4 ± 0.7	25.5 ± 0.5
FeOx-10	20.0 ± 0.3	23.1 ± 0.6
FeOx-21	30.5 ± 0.4	33.9 ± 0.4

## Supplementary Materials

The following are available online at http://www.mdpi.com/1996-1944/11/9/1654/s1, Figure S1. High resolution TEM imagines, Figure S2. TGA curves of all core-shell nanoparticles samples, Figure S3. DLS size distribution histograms of core-shell nanoparticles, Figure S4. DLS-heating curves of free polymer dispersions in water at different concentration, Figure S5. DLS-heating curves of FeOx-5 dispersions in water at different concentration, Figure S6. DLS-heating curves of FeOx-7 dispersions in water at different concentration, Figure S7. DLS-heating curves of FeOx-10 dispersions in water at different concentration, Figure S8. DLS-heating curves of FeOx-21 dispersions in water at different concentration, Figure S9. DSC curve fittings of free polymer (26 kgmol^−1^) at different concentrations, Figure S10. DSC curve fittings of FeOx-5 at different concentrations at different concentrations, Figure S11. DSC curve fittings of FeOx-7 at different concentrations at different concentrations, Figure S12. DSC curve fittings of FeOx-10 at different concentrations at different concentrations, Figure S13. DSC curve fittings of FeOx-21 at different concentrations at different concentrations.
