# Supplementary material for: Thermoresponsive Core-Shell Nanoparticles: Does Core Size Matter?
Source: Materials (Basel). 2018 Sep 7;11(9):1654. doi: 10.3390/ma11091654 (PMC6163620; doi:10.3390/ma11091654)
Supplement: Supplementary file 1 [file materials-11-01654-s001.pdf]

materials

Article

Thermoresponsive core-shell nanoparticles: does core size matter?

Martina Schroffenegger[1] and Erik Reimhult[1,*]

[1] University of Natural Resources and Life Sciences Vienna, Muthgasse 11, 1190 Vienna, Austria; martina.schroffenegger@boku.ac

* Correspondence: erik.reimhult@boku.ac.at; Tel.: +43-1-47654-80211

Received: date; Accepted: date; Published: date

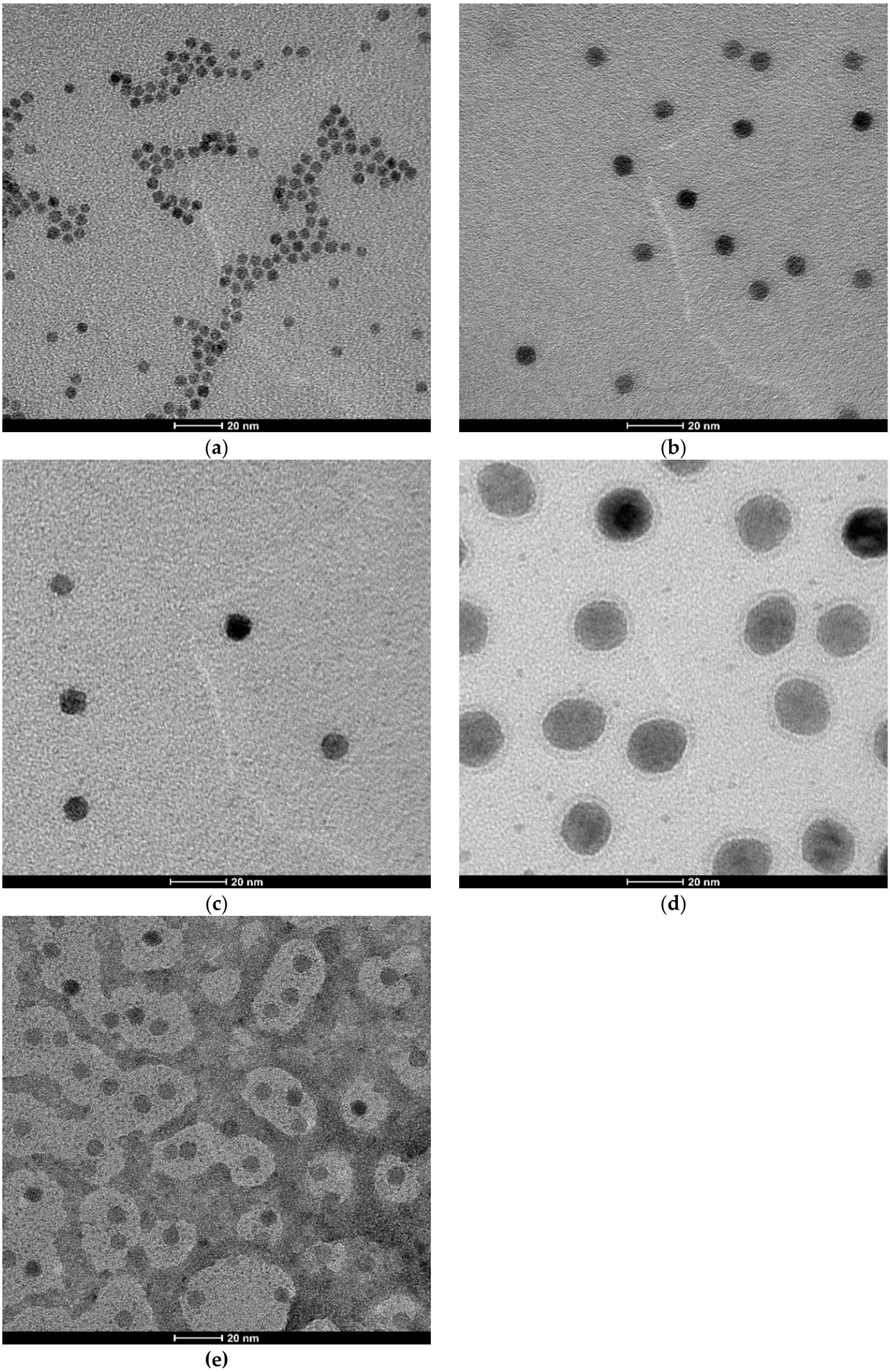

Figure S1: High resolution TEM imagines: (**a**) FeOx-5, (**b**) FeOx-7, (**c**) FeOx-10, (**d**) FeOx-21 and (e) FeOx-7 imaged by negative background staining to visualize the dried shell.

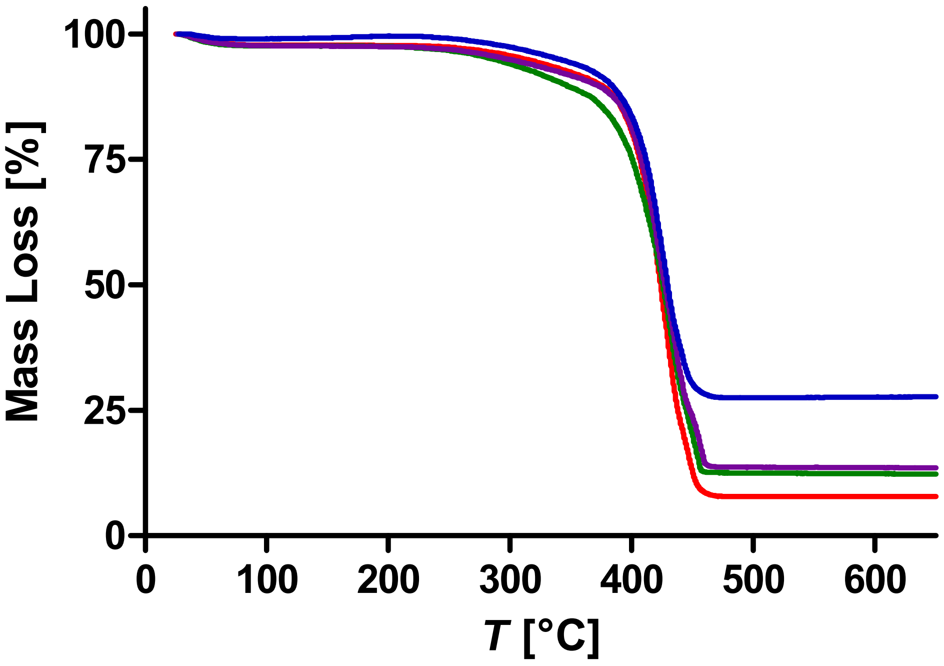

Figure S2. TGA curves of all core-shell nanoparticles samples. TGA was measured with a heating rate of 10 °C min^{-1} at a constant flow of 80 mL min^{-1} of synthetic air. Red: FeOx-5, green: FeOx-7, purple: FeOx-10, blue: FeOx-21.

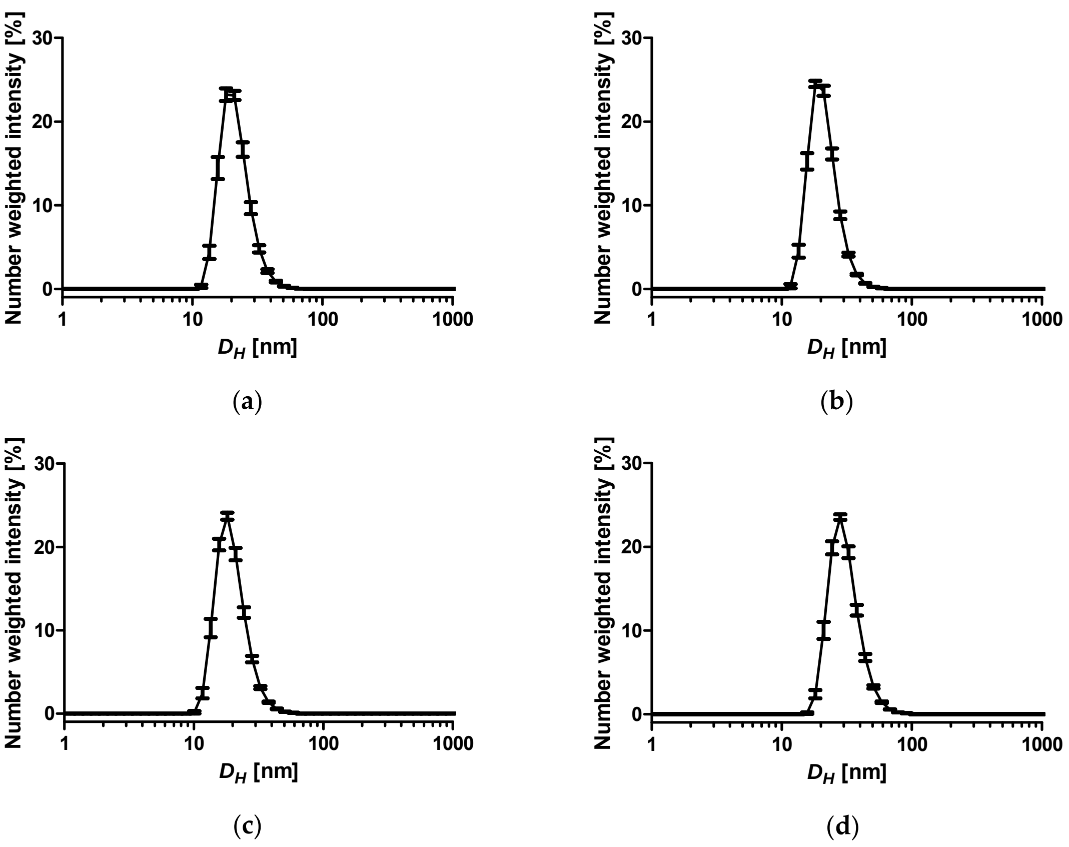

Figure S3: DLS particle distribution histograms of core-shell nanoparticles: (**a**) FeOx-5, (**b**) FeOx-7, (**c**) FeOx-10 and (**d**) FeOx-21. The presented data is a sum of all measurements performed at temperatures below the CFT.

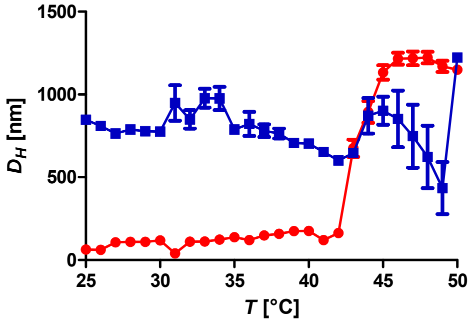

(**a**)

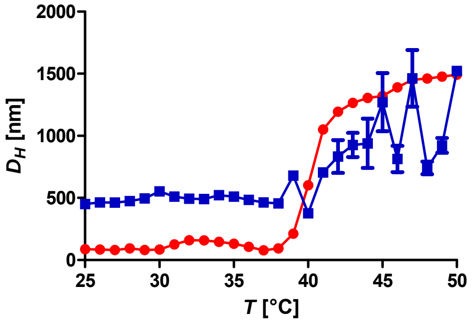

(**b**)

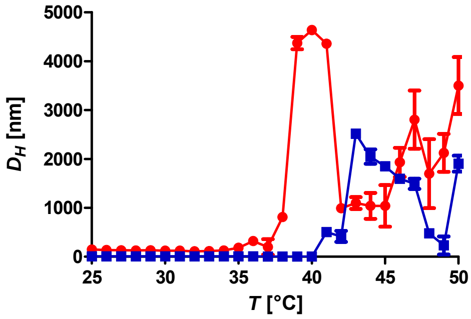

(**c**)

Figure S4. DLS-heating curves of free polymer dispersions in water at different concentration: (**a**): 0.1, (**b**): 1 and (**c**): 10 g L^{-1}. Red: heating curve, blue: cooling curve. Mean values and standard error of the number weighted diameter were calculated from three measurements for each temperature step.

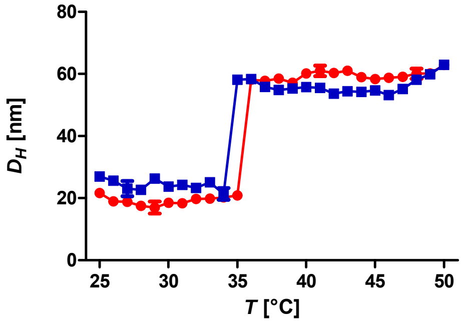

(**a**)

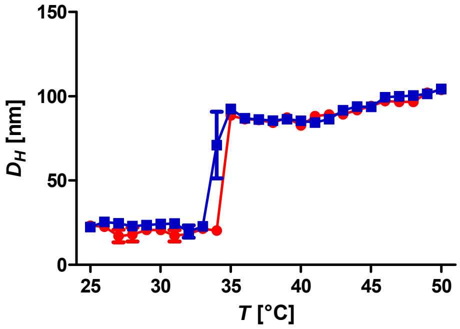

(**b**)

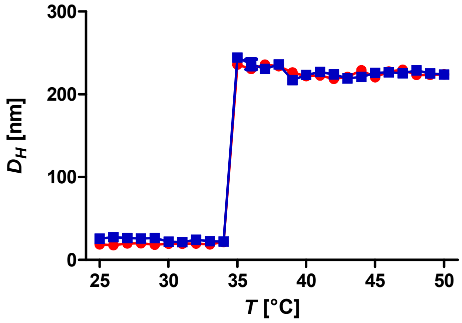

(**c**)

Figure S5. DLS-heating curves of FeOx-5 dispersions in water at different concentration: (**a**): 0.1, (**b**): 1 and (**c**): 10 g L^{-1}. Red: heating curve, blue: cooling curve. Mean values and standard error of the number weighted diameter were calculated from three measurements for each temperature step.

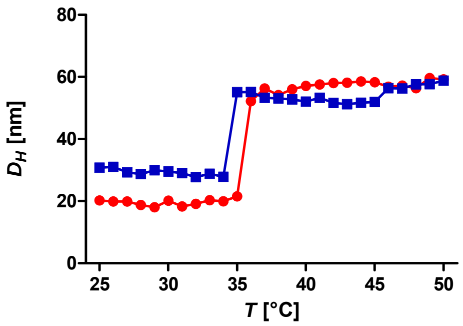

(**a**)

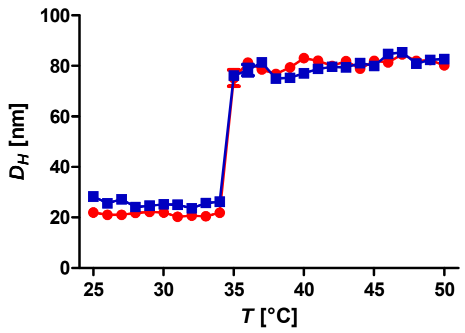

(**b**)

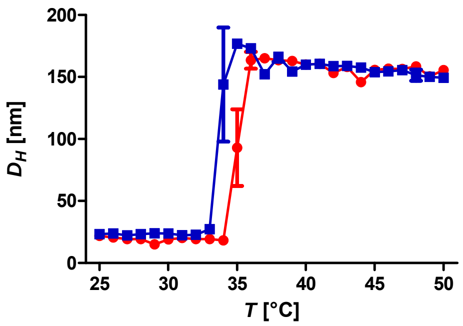

(**c**)

Figure S6. DLS-heating curves of FeOx-7 dispersions in water at different concentration: (**a**): 0.1, (**b**): 1 and (**c**): 10 g L^{-1}. Red: heating curve, blue: cooling curve. Mean values and standard error of the number weighted diameter were calculated from three measurements for each temperature step.

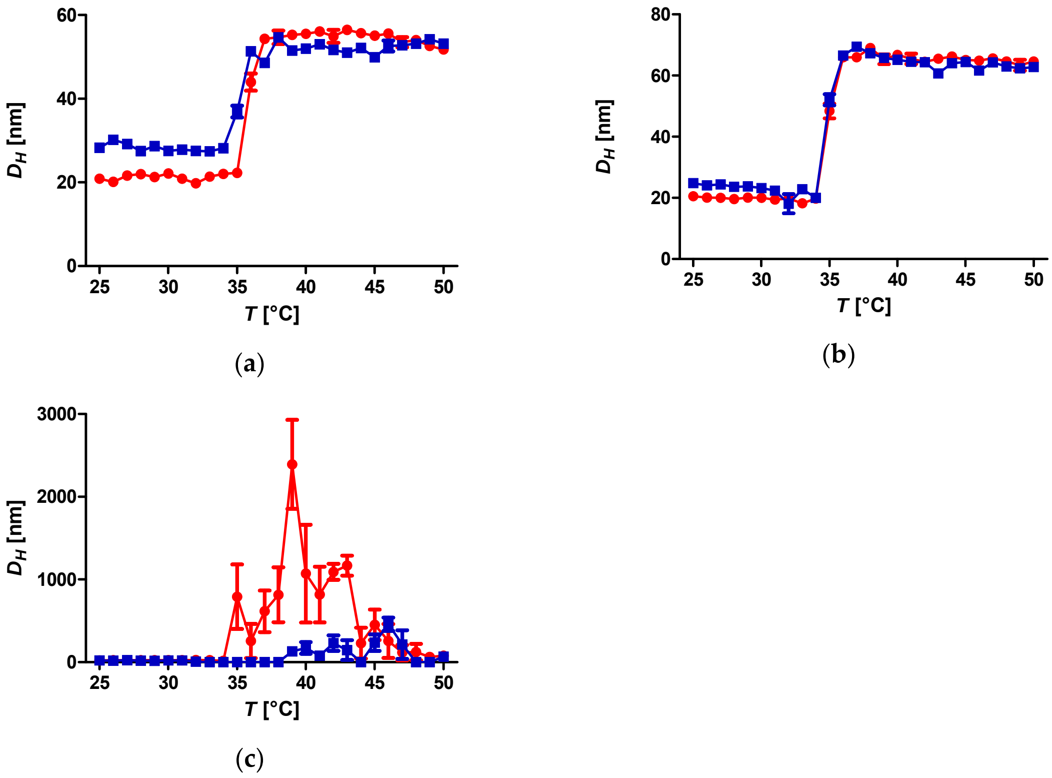

Figure S7. DLS-heating curves of FeOx-10 dispersions in water at different concentration: (**a**): 0.1, (**b**): 1 and (**c**): 10 g L^{-1}. Red: heating curve, blue: cooling curve. Mean values and standard error of the number weighted diameter were calculated from three measurements for each temperature step.

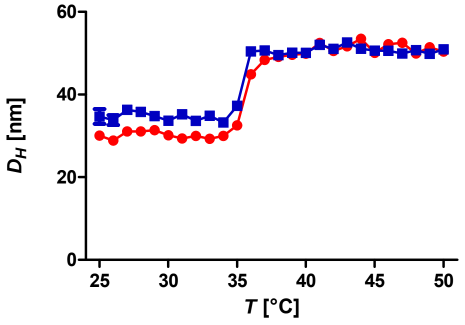

(**a**)

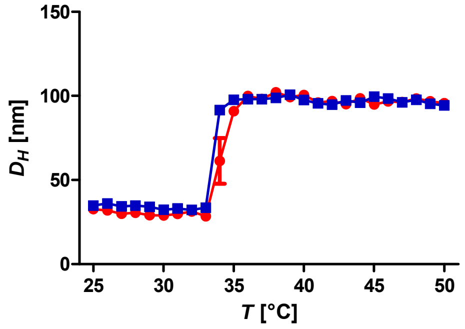

(**b**)

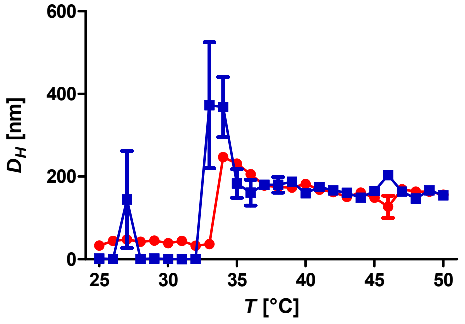

(**c**)

Figure S8. DLS-heating curves of FeOx-21 dispersions in water at different concentration: (**a**): 0.1, (**b**): 1 and (**c**): 10 g L^{-1}. Red: heating curve, blue: cooling curve. Mean values and standard error of the number weighted diameter were calculated from three measurements for each temperature step.

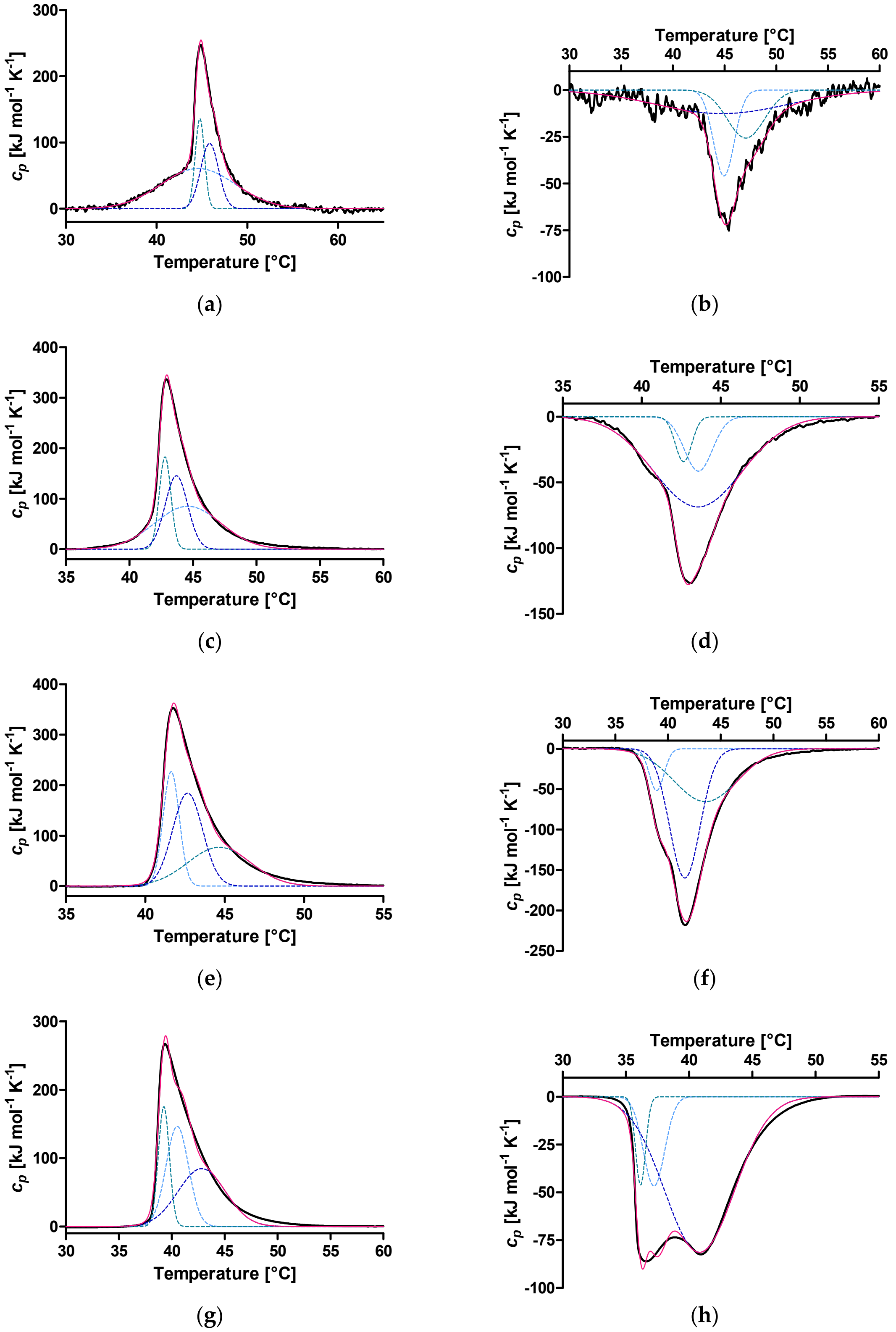

Figure S9. DSC curve fittings of free polymer (26 kgmol^{-1}) at different concentrations: (**a**) and (**b**) at a concentration of 0.1 g L$^{-1,}$ (**c**) and (**d**) at a concentration of 0.5 g L^{-1}, (**e**) and (**f**) at a concentration of 1 g L^{-1} and (**g**) and (**h**) at a concentration of 10 g L^{-1}. The samples were measured in Milli-Q water with a heating rate of 1 °C min^{-1}. Left: heating curves, right: cooling curves. Black: raw data of the measurements, dashed lines: fitted curves, pink: sum of fitted curves.

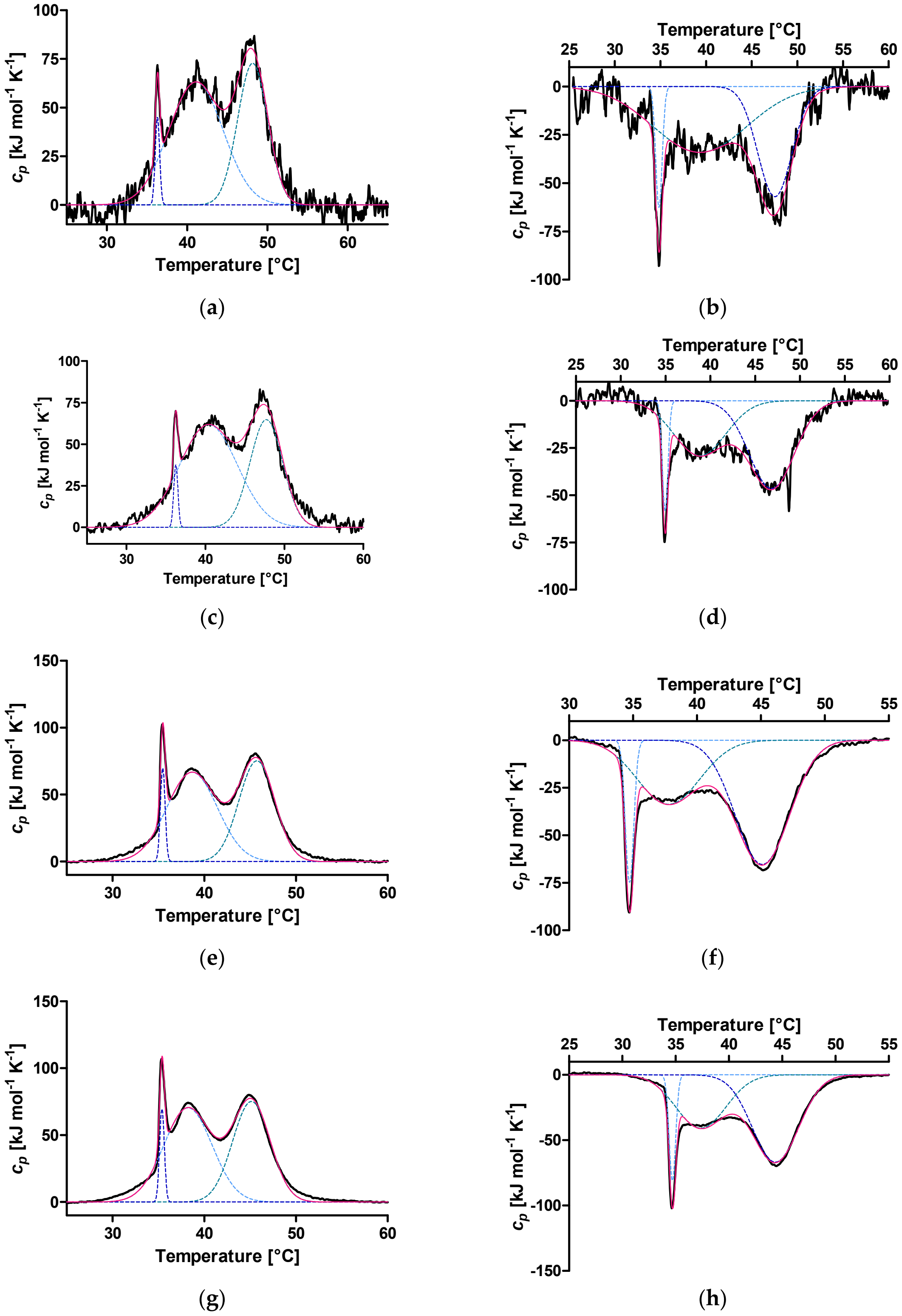

c_p [kJ mol-1 K-1]
Temperature [°C]
(a)
(b)
(c)
(d)
(e)
(f)
(g)
(h)

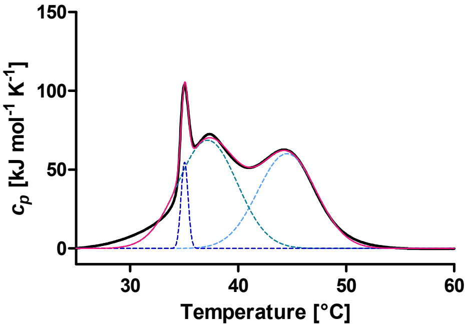

(**i**)

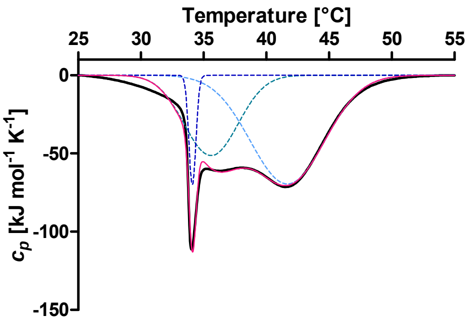

(**j**)

Figure S10. DSC curve fittings of FeOx-5 at different concentrations at different concentrations: (**a**) and (**b**) at a concentration of 0.07 g $L^{-1,}$ (**c**) and (**d**) at a concentration of 0.11 g L^{-1}, (**e**) and (**f**) at a concentration of 0.54 g L^{-1}, (**g**) and (**h**) at a concentration of 1.09 g L^{-1} and (**i**) and (**j**) at a concentration of 10.9 g L^{-1}. The samples were measured in Milli-Q water with a heating rate of 1 °C min^{-1}. Left: heating curves, right: cooling curves. Black: raw data of the measurements, dashed lines: fitted curves, pink: sum of fitted curves.

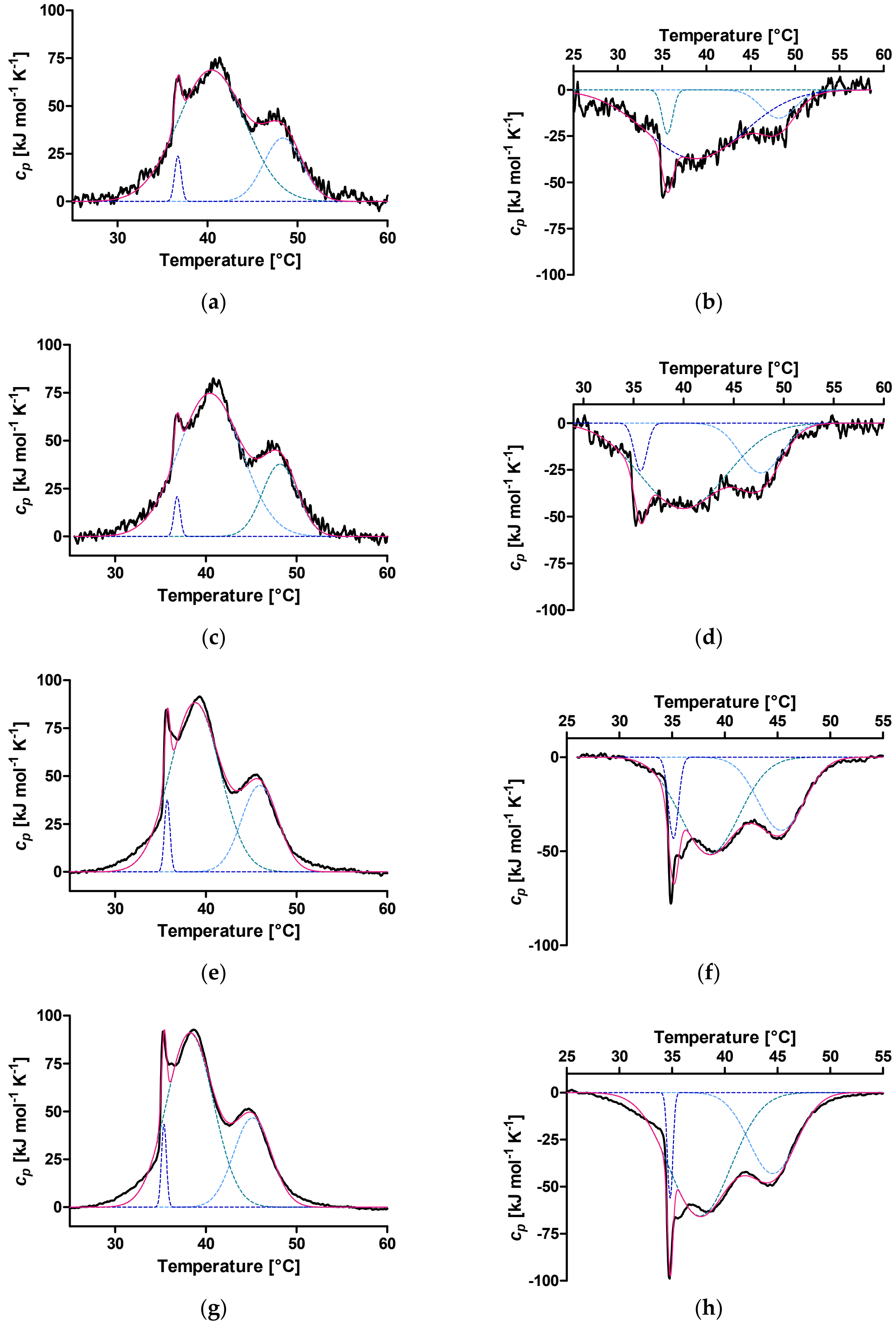

cp [kJ mol-1 K-1]
Temperature [°C]
(a)
(b)
(c)
(d)
(e)
(f)
(g)
(h)

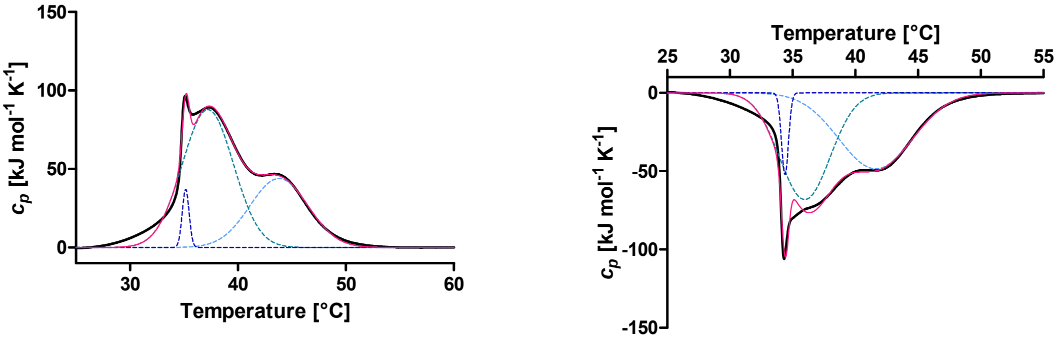

(**i**) (**j**)

Figure S11. DSC curve fittings of FeOx-7 at different concentrations at different concentrations: (**a**) and (**b**) at a concentration of 0.11 g $L^{-1,}$ (**c**) and (**d**) at a concentration of 0.14 g L^{-1}, (**e**) and (**f**) at a concentration of 0.57 g L^{-1}, (**g**) and (**h**) at a concentration of 1.14 g L^{-1} and (**i**) and (**j**) at a concentration of 10.9 g L^{-1}. The samples were measured in Milli-Q water with a heating rate of 1 °C min^{-1}. Left: heating curves, right: cooling curves. Black: raw data of the measurements, dashed lines: fitted curves, pink: sum of fitted curves.

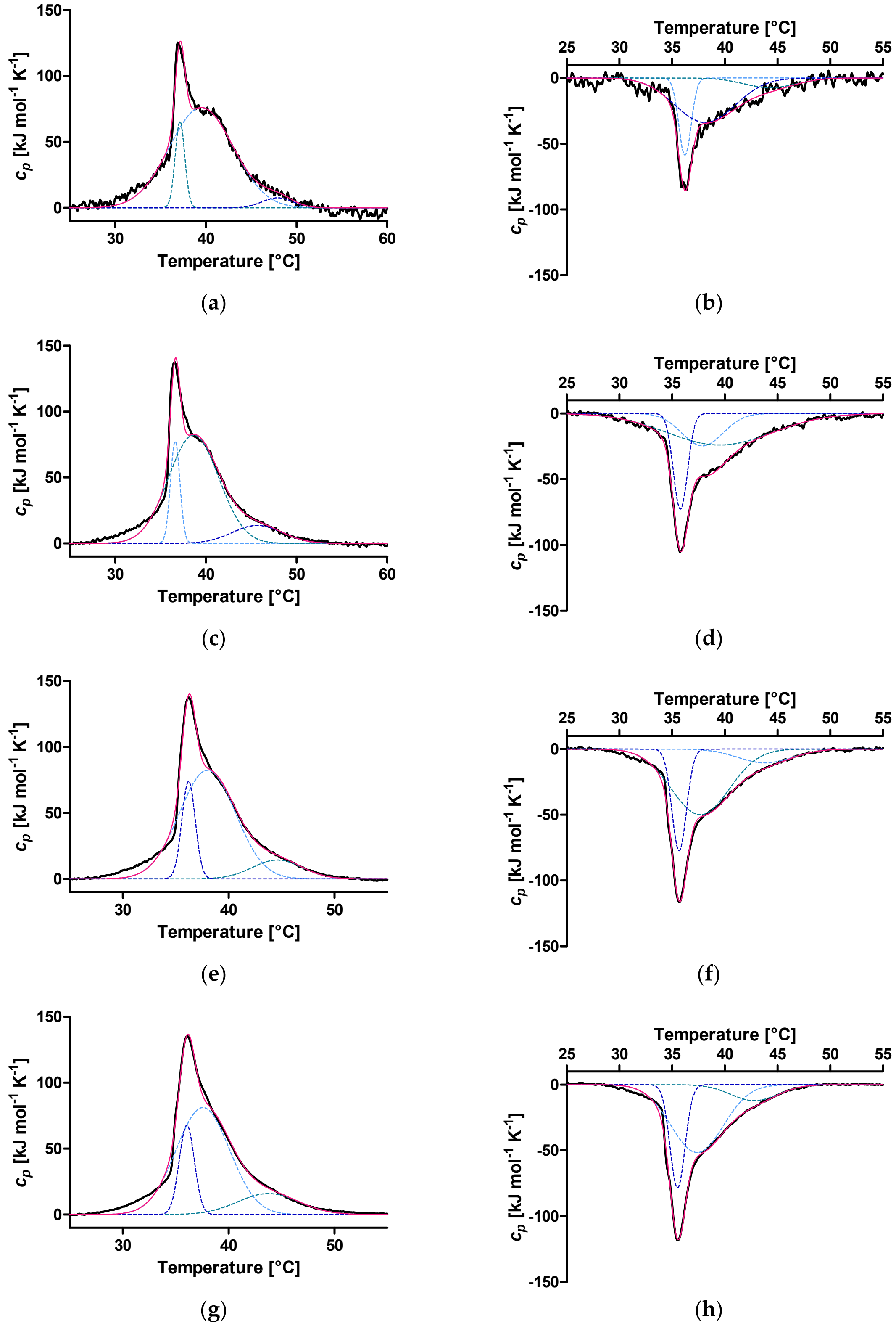

cp [kJ mol-1 K-1]
Temperature [°C]
150
100
50
0
-50
-100
-150
25
30
35
40
45
50
55
60
(a)
(b)
(c)
(d)
(e)
(f)
(g)
(h)

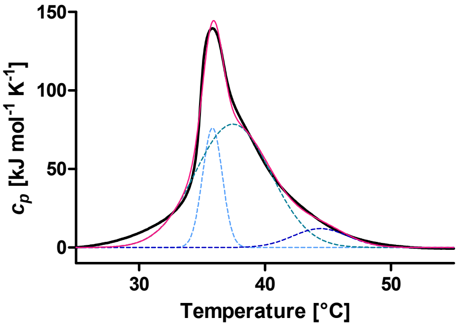

(**i**)

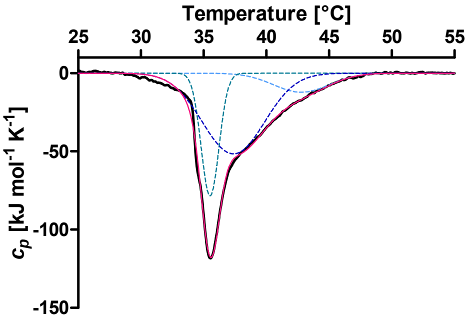

(**j**)

Figure S12. DSC curve fittings of FeOx-10 at different concentrations at different concentrations: (**a**) and (**b**) at a concentration of 0.12 g L$^{-1,}$ (**c**) and (**d**) at a concentration of 0.23 g L^{-1}, (**e**) and (**f**) at a concentration of 0.58 g L^{-1}, (**g**) and (**h**) at a concentration of 1.16 g L^{-1} and (i) and (j) at a concentration of 11.6 g L^{-1}. The samples were measured in Milli-Q water with a heating rate of 1 °C min^{-1}. Left: heating curves, right: cooling curves. Black: raw data of the measurements, dashed lines: fitted curves, pink: sum of fitted curves.

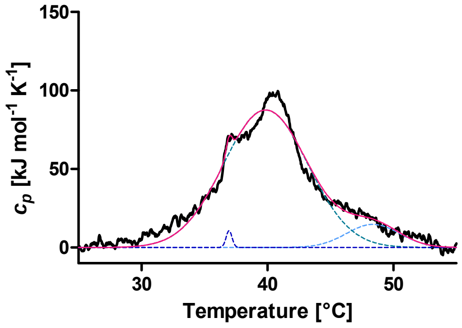

(**a**)

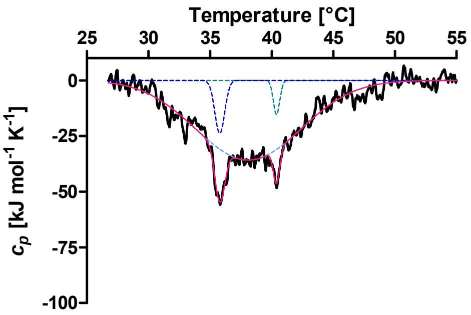

(**b**)

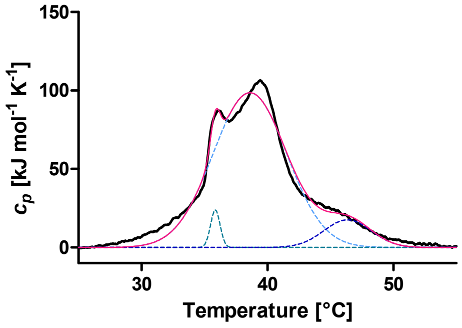

(**c**)

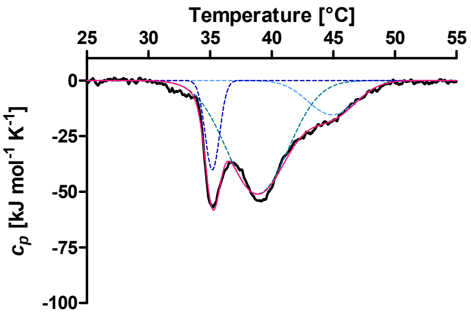

(**d**)

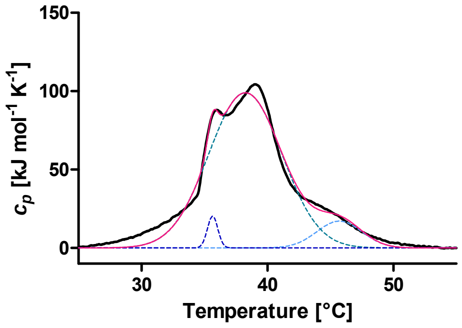

(**e**)

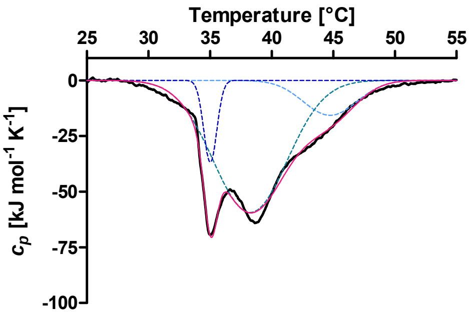

(**f**)

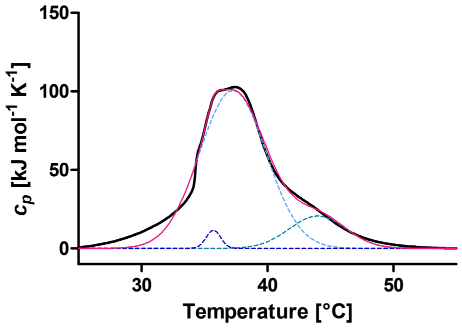

(**g**)

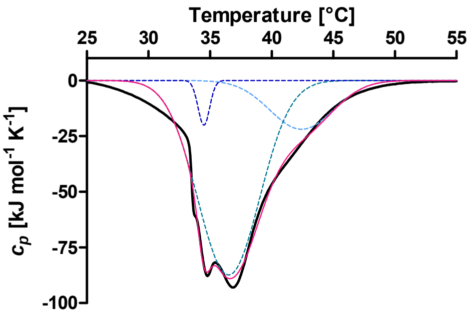

(**h**)

Figure S13. DSC curve fittings of FeOx-21 at different concentrations at different concentrations: (**a**) and (**b**) at a concentration of 0.14 g L$^{-1,}$ (**c**) and (**d**) at a concentration of 0.70 g L^{-1}, (**e**) and (**f**) at a concentration of 1.39 g L^{-1} and (**g**) and (**h**) at a concentration of 13.9 g L^{-1}. The samples were measured in Milli-Q water with a heating rate of 1 °C min^{-1}. Left: heating curves, right: cooling curves. Black: raw data of the measurements, dashed lines: fitted curves, pink: sum of fitted curves.

© 2018 by the authors. Submitted for possible open access publication under the terms and conditions of the Creative Commons Attribution (CC BY) license (http://creativecommons.org/licenses/by/4.0/).
